# Molecular Background of Pi Deficiency-Induced Root Hair Growth in *Brassica carinata* – A Fasciclin-Like Arabinogalactan Protein Is Involved

**DOI:** 10.3389/fpls.2018.01372

**Published:** 2018-09-19

**Authors:** Thomas W. Kirchner, Markus Niehaus, Kim L. Rössig, Timo Lauterbach, Marco Herde, Helge Küster, Manfred K. Schenk

**Affiliations:** ^1^Institute of Plant Nutrition, Leibniz Universität Hannover, Hanover, Germany; ^2^Institute of Plant Genetics, Leibniz Universität Hannover, Hanover, Germany

**Keywords:** CRISPR/Cas9, BcFLA1, hairy root, K deficiency, local and systemic response, MACE, N deficiency, split-root

## Abstract

Formation of longer root hairs under limiting phosphate (P) conditions can increase the inorganic P (Pi) uptake. Here, regulatory candidate genes for Pi deficiency-induced root hair growth were identified by comparison of massive analysis of cDNA ends (MACE) provided expression profiles of two *Brassica carinata* cultivars (cv.) differing in their root hair response to Pi deficiency: cv. Bale develops longer root hairs under Pi deficiency, but not cv. Bacho. A split-root experiment was conducted for the differentiation between locally and systemically regulated genes. Furthermore, plants were exposed to nitrogen and potassium deficiency to identify P-specific reacting genes. The latter were knocked out by CRISPR/Cas9 and the effect on the root hair length was determined. About 500 genes were differentially expressed under Pi deficiency in cv. Bale, while these genes did not respond to the low P supply in cv. Bacho. Thirty-three candidate genes with a potential regulatory role were selected and the transcriptional regulation of 30 genes was confirmed by quantitative PCR. Only five candidate genes seemed to be either exclusively regulated locally (two) or systemically (three), whereas 25 genes seemed to be involved in both local and systemic signaling pathways. Potassium deficiency affected neither the root hair length nor the expression of the 30 candidate genes. By contrast, both P and nitrogen deficiency increased the root hair length, and both affected the transcript levels in 26 cases. However, four genes reacted specifically to Pi starvation. These genes and, additionally, *INORGANIC PHOSPHATE TRANSPORTER 1* (*BcPHT1*) were targeted by CRISPR/Cas9. However, even if the transcript levels of five of these genes were clearly decreased, *FASCICLIN-LIKE ARABINOGALACTAN PROTEIN 1* (*BcFLA1*) was the only gene whose downregulation reduced the root hair length in transgenic hairy roots under Pi-deficient conditions. To the best of our knowledge, this is the first study describing a fasciclin-like arabinogalactan protein with a predicted role in the Pi deficiency-induced root hair elongation.

## Introduction

Since phosphate (P) in the soil available to plants is often limited, plants rely on mechanisms increasing the inorganic P (Pi) uptake (Vance et al., 2003). One of these is the development of longer root hairs under Pi deficiency, which increases the depletion zone around the root and, thus, the acquisition capability (Jungk, 2001). Among the root hair development steps, the root hair elongation, during which the root hair grows by tip growth, is decisive for the final root hair size (Datta et al., 2011). The general root hair elongation mechanisms are, at least in *Arabidopsis thaliana*, relatively well known, but the regulating mechanisms underlying the P deficiency-induced root hair elongation are still not completely understood.

Recent studies in Arabidopsis revealed that transcription factors play an important role in the root hair elongation under Pi starvation. The bHLH transcription factor ROOT HAIR DEFECTIVE 6 induces the expression of another bHLH transcription factor, ROOT HAIR DEFECTIVE 6-LIKE 4 (RSL4), which, in turn, regulates genes needed for cell growth (Yi et al., 2010; Vijayakumar et al., 2016). Interestingly, RSL4 synthesis under Pi deficiency and, therefore, its presence in the root hair was prolonged, so that the root hair growth duration and, accordingly, the final root hair size was enhanced (Datta et al., 2015). Moreover, some of the target genes of RSL4 can be upregulated by ETHYLENE INSENSITIVE 3, which is a key transcription factor in the ethylene signaling pathway that is upregulated by Pi deficiency, thus, also increasing the root hair length (Song et al., 2016). In addition, there is a functional paralog of *RSL4*, *LOTUS JAPONICUS ROOTHAIRLESS1-LIKE 3* (*LRL3*), which was upregulated in response to Pi deficiency and shown to be necessary for a proper root hair elongation (Bruex et al., 2012; Salazar-Henao et al., 2016; Salazar-Henao and Schmidt, 2016). Furthermore, the homeodomain transcription factor *ALFIN-LIKE 6* controls the transcription of several genes involved in root hair elongation under Pi deficiency (Chandrika et al., 2013).

In addition to transcription factors, protein kinases also seem to have key roles during root hair elongation under Pi deficiency, since many protein kinases which were regulated in response to Pi starvation were associated with root hair growth in a co-expression analysis (Lan et al., 2013). Phosphatidylinositol phosphate 5-kinases (*PIP5K*s) were identified to promote root hair elongation in response to Pi starvation (Wada et al., 2015). The *PIP5K*s produce a signaling peptide, phosphatidylinositol 4,5-bisphosphate, and the authors concluded that, at least at young seedling stages, the *PIP5K* genes are responsible for transferring the Pi deficiency signal to the root hair elongation pathway. However, the authors predicted signaling pathways independent of the *PIP5K* genes playing a role during prolonged Pi starvation.

Moreover, calcium signaling and plant RHO GTPases (ROPs) seem to be involved in the Pi deficiency-induced root hair elongation, since a calcium-binding EF-hand family protein (CALMODULIN-LIKE 25, CML25) and ROP GUANINE NUCLEOTIDE-EXCHANGE FACTOR 4 (ROPGEF4) seem to regulate the Pi deficiency-induced root hair elongation in Arabidopsis negatively (Lin et al., 2011). The ROPGEF4, in cooperation with the receptor-like kinase FERONIA (FER), plays a role in the activation of ROPs (Duan et al., 2010).

In spite of the identification of several players involved in the Pi deficiency-induced root hair elongation, especially the network of regulatory genes, which may act at the beginning of the signal transduction cascade leading to the longer root hairs under Pi deficiency, still exhibits major knowledge gaps. Therefore, the aim of the present study was to identify and functionally characterize new candidate genes for the regulation of the Pi deficiency-induced root hair elongation.

Preliminary studies revealed two cultivars (cv.) of *Brassica carinata* (Ethiopian mustard) with a difference in their Pi uptake efficiency (Eticha and Schenk, 2001). Cultivar Bale develops longer root hairs under Pi deficiency compared to sufficient Pi conditions, whereas cv. Bacho develops short root hairs under both conditions. We used massive analysis of cDNA ends (MACE) to compare expression profiles of the *B. carinata* cv.s under both sufficient and deficient Pi conditions. The MACE provides a high resolution, since one single cDNA fragment is analyzed per transcript molecule, consequently, medium and low level transcripts can also be detected (Kahl et al., 2012). We identified around 500 genes in two different annotations with a regulation that indicated a role in the root hair elongation under Pi deficiency. More than 30 of these were investigated further, because of a potential regulatory function, and the regulation of nearly all of them could be validated by quantitative PCR (qPCR). A split-root experiment showed that most of these genes may be involved in both local and systemic signaling pathways. Furthermore, a few genes were upregulated exclusively under Pi deficiency, although both Pi and nitrogen (N) deficiency increased root hair length in cv. Bale. One of these genes was *FASCICLIN-LIKE ARABINOGALACTAN PROTEIN 1* (*BcFLA1*), whose downregulation by CRISPR/Cas9 in a hairy root system led to a reduced root hair length compared to the wildtype. The role of *BcFLA1* in Pi deficiency signaling is discussed.

## Materials and Methods

### Plant Material and Growth Conditions

#### MACE Experiment (1st Experiment)

Seeds of *B. carinata* cv. Bale and cv. Bacho were surface sterilized for 1 min in 1% sodium hypochlorite, put on 0.7% (w/v) phyto agar (pH 5.8; Duchefa Biochemie, Haarlem, Netherlands) and then stratified for 3 days in the dark at 4°C for the MACE experiment. Afterward, the seeds were germinated *in vitro* for five (Bale) and 6 days (Bacho), respectively, with a day/night interval of 16/8 h at 23°C. The plants were then transferred into a climate chamber (photoperiod, 16/8 h light/dark; temperature, 20/15°C day/night; relative humidity: 75%; light intensity: 220 μmol m^-2^ s^-1^) and cultivated in nutrient solutions containing (mM) 2.25 Ca(NO_3_)_2_ × 4 H_2_O, 2.5 K_2_SO_4_, 1 MgSO_4_ × 7 H_2_O, 0.25 KCl and exclusively in the control treatment 1 KH_2_PO_4_. Furthermore, the nutrient solutions contained the following micronutrient concentrations (μM): 25 H_3_BO_3_, 1.5 MnSO_4_, 1.5 ZnSO_4_, 0.5 CuSO_4_, 0.025 (NH_4_)_6_Mo_7_O_24_ and 35.8 Fe (Fe^III^-EDTA). The pH was adjusted to 5.3 and readjusted during the cultivation as needed. After 8 days, root tips with a length of 2 cm were cut for later MACE and qPCR analysis, frozen immediately in liquid nitrogen and then stored at -85°C. The root tips of 42 plants (two root tips per plant) were pooled for each treatment. Additionally, six replications, each consisting of seven plants (four root tips per plant), were harvested from the same plants. Before cutting the root tips, the roots were washed in diethylpyrocarbonate (DEPC) twice to minimize the contamination with RNAses. Furthermore, total shoots for dry matter (dm) and P determination were harvested and dried for 3 days at 60°C (four replications of four shoots each). Eight complete roots per treatment were incubated for 1 h in 70% (v/v) EtOH and then stored in tap water at 4°C for later root hair length measurement.

#### Independently Replicated (2nd) Experiment

The independently replicated experiment was identical to the MACE experiment, but 30 root tips were harvested per replication (six plants of five root tips each) for the expression analysis. Furthermore, six replications of three shoots each (for dm and P determination) and 12 total roots (for root hair length measurement) were harvested per treatment.

#### Split-Root Experiment

*Brassica carinata* cv. Bale plants were germinated as before and then hydroponically grown under the conditions above and in the above nutrient solution containing 0.05 mM KH_2_PO_4_ for 5 days for the split-root experiment. The main root was then cut and 1 day later, the plants were transferred into a split-root system consisting of nutrient solutions with (+ +) or without (--) 1 mM Pi on both sides and without Pi only on one side and sufficient (1 mM) Pi on the other side (+ -). After the appearance of P deficiency symptoms at the shoot and a distinct increase of the root hair length in the [--] plants, root tips (three replications consisting of ten plants each, of each plant two root tips were taken for expression analysis) and 30 total roots (+ + and --) and root halves (+ -), respectively (for root hair length measurement), were harvested and treated as before. Additionally, three replications of 21 shoots each and three replications of five total roots each (+ + and --) and five root halves each (+ -), respectively, were harvested for dm and P determination.

#### Nutrient Specificity Experiment

Seeds of *B. carinata* cv. Bale and Bacho were germinated between filter papers under the same climate chamber conditions as before for the nutrient specificity experiment. The composition of the nutrient solutions was the same as before for the control and Pi deficiency treatment, but Ca(NO_3_)_2_ × 4 H_2_O was substituted by CaCl_2_ × 2 H_2_O in the N deficiency treatment and K_2_SO_4_ and KCl were omitted and KH_2_PO_4_ was substituted by NaH_2_PO_4_ × 2 H_2_O in the potassium (K) deficiency treatment. After 8 days, root tips (six replications of six plants each of five root tips each for expression analysis), shoots (six replications of three shoots each for dm and P determination) and 12 complete roots (for root hair length measurement) were harvested and treated as before.

#### CRISPR Experiments

Plant cultivation and screening for transgenic roots was done as described in Kirchner et al. (2017) for the CRISPR experiments. As many 2-cm root tips as possible were harvested from each transgenic root for expression analysis. The remaining transgenic roots were used for the verification of the gene editing.

### Determination of the Nutrient Concentrations

An amount of 50 mg dm was dry-ashed overnight at 480°C, dissolved in 1 mL 1:3 diluted HNO_3_, diluted with 9 mL H_2_O and filtrated (Rotilabo^®^-round filters, type 15A) for the extraction of total P and K.

The Pi was extracted, according to Grunwald et al. (2009), using 10 mg (shoot) and 5 mg (root) dm.

Both total P and Pi concentrations were photometrically determined, according to Gericke and Kurmies (1952).

Shoot P and K concentration in the nutrient specificity experiment were determined by inductively coupled plasma – mass spectrometry (ICP-MS) (Agilent 7500 cx, Agilent Technologies, Santa Clara, CA, United States). Shoot N concentration was determined by an elemental analyzer (Vario EL *III*, Elementar, Langenselbold, Germany).

### Root Hair Length Measurement

Roots were stained in 0.05% (w/v) toluidine blue for 1–2 h. Subsequently, two 2-cm root tips were cut per plant, put into tap water and examined by the MZ10 F (Leica Mikrosysteme Vertrieb GmbH, Wetzlar, Germany). Root tips were pulled over 0.3% (w/v) agar–agar, until the root hairs were straight. Pictures were taken from the root sections with fully elongated root hairs and the root hair length was measured. For the nutrient specificity and CRISPR experiments pictures were taken using the ‘SMZ25’ (Nikon, Düsseldorf, Germany) and root hair length was measured using the corresponding software ‘NIS-Elements’ instead. Root tips of the transgenic first and second order lateral roots were cut for the CRISPR experiments.

### RNA Isolation

Frozen pool samples were homogenized by mortar and pestle. The RNA was then isolated using the NucleoSpin miRNA kit (MACHEREY-NAGEL GmbH & Co., KG, Düren, Germany), following the manufacturer’s instructions (using the large RNA isolation procedure).

The frozen samples of the biological replications were homogenized in a swing mill at 28 s^-1^ for 2 min (MM 400, Retsch GmbH, Haan, Germany). The RNA was isolated using the GENEzol^TM^ reagent (Geneaid Biotech Ltd., New Taipei City, Taiwan) (MACE, 2nd experiment and split-root experiment) and the peqGOLD TriFast^TM^ reagent (VWR International GmbH, Darmstadt) (nutrient specificity and CRISPR experiments), respectively, according to the manufacturer’s instructions.

The RNA Quality was electrophoretically tested on a 1% (w/v) non-denaturating agarose gel containing 0.004% (v/v) Midori Green Advance (Nippon Genetics Europe, Dueren, Germany) and a runtime of 35 min at 6.9–7.6 V cm^-1^ in Tris-acetate-EDTA (TAE) buffer. The RNA quantity was determined using the NanoPhotometer^®^ P-Class P 300 (Implen, Munich, Germany).

### Massive Analysis of cDNA Ends (MACE)

One isolated RNA pool sample with a total amount of 6–12.7 μg and a concentration between 280 and 550 ng μL^-1^ for each treatment was used for MACE (GenXPro, Frankfurt am Main, Germany, according to Kahl et al., 2012).

The tags of the MACE were annotated to two related plant species. The first annotation process was done by GenXPro in which the tags were annotated against the genome and transcriptome of *Brassica rapa* (Ensembl, annotation release 20, Kersey et al., 2016), whereby non-annotatable tags were assembled *de novo* using TrinityRNASeq (version: trinityrnaseq_r20140717) and CAP3 (version date: 12/21/07). Consensus sequences were generated against the reference sequences (*B. rapa* genome and transcriptome and *de novo* assembly) using NovoAlign (V3.00.05). The second annotation process was performed with CLC Genomics Workbench (version 7.5.1; CLC Bio, Aarhus, Denmark). Firstly, the tags were trimmed in two steps using a 50 and subsequently a 25 bp poly-A adapter as template. Simultaneously, tags were trimmed to a quality limit of a maximum of 0.05 and to contain less than two ambiguous bases. Tags shorter than 50 bp were discarded. The tags were then annotated against *Brassica napus* (*Brassica napus* gene index (BnGI) release 5) using the RNAseq tool (settings: similarity fraction = 0.8, length fraction = 0.8, mismatch cost = 2, insertion cost = 3, deletion cost = 3) and consensus sequences were generated from the annotated tags.

The complete MACE dataset is accessible in the sequence and read archive under the accession number SRP124702.

A Gene Ontology (GO) enrichment analysis was performed using the data from the *Brassica rapa* annotation. Significantly enriched GO terms were determined by Fisher’s exact test (*p* < 0.01). Redundant GO terms were removed using REVIGO (Supek et al., 2011), whereby ‘medium’ (0.7) was selected for the resulting list and *Arabidopsis thaliana* was chosen as database.

### Candidate Gene Selection

The list of annotated genes was filtered automatically using the criteria twofold regulation in Bale (I), at least three normalized tags in Bale (II), a regulation in Bacho between 0.7 and 1.5 (III), and the number of normalized tags in the Pi deficiency treatment had to be at least 1.5-fold higher (upregulated genes) or lower (downregulated genes) in Bale than in Bacho (IV) (**Supplementary Table 1**). Only genes with a potential regulatory function were chosen from the resulting list for the subsequent analyses. Additionally, *BcPIP5K1* was selected from the MACE dataset, because Wada et al. (2015) uncovered a role in the Pi deficiency-induced root hair elongation for its counterpart in *Arabidopsis thaliana*, *AtPIP5K3*.

The candidate gene sequences obtained by MACE and 5′ RNA ligase-mediated rapid amplification of cDNA ends (5′ RLM-RACE) were submitted to the NCBI database under the accession numbers listed in **Supplementary Table 2**.

### Expression Analysis

The cDNA synthesis and qPCR were performed, as described in Kirchner et al. (2017). Gene-specific primer pairs were designed on the consensus sequence of each candidate gene obtained by MACE in regions with high similarity between the four treatments (**Supplementary Table 3**). *LOTUS JAPONICUS ROOTHAIRLESS1-LIKE 3-LIKE (BcLRL3L)* primers were designed to bind both *BcLRL3L1* and *2*, whereby there was one mismatch in the reverse primer for *BcLRL3L2* at position 16. Primers for *INDUCED BY Pi STARVATION 2 (AtIPS2)* (Shin et al., 2006) were designed on the corresponding Arabidopsis sequence (AT5G03545).

Primers with an efficiency between 85 and 115% were used for expression analysis if the discrepancy in the efficiency of the two cv.s was 10% at the most.

*UBIQUITIN CONJUGATING ENZYME 9* (*AtUBC9*; AT4G27960) was used as an endogenous control in all the experiments, because of its invariant expression under nutrient stress conditions (Czechowski et al., 2005). *ELGONGATION FACTOR 1 ALPHA 1* (*BcEF-1-a1*) was used as an additional control in the nutrient specificity experiment, whereupon the geometric mean of both endogenous controls was calculated and compared to the target genes.

The annealing temperature (T_a_) was consistently 60°C in all qPCR runs.

Statistics were performed according to Steibel et al. (2009), using the R package ‘qpcrmix’.

### Knockout of the Candidate Genes by CRISPR/Cas9

#### Construct Preparation

The construct preparation including the guide RNA (gRNA) design was carried out, as described in Kirchner et al. (2017). Briefly, the Bale gene sequence of selected candidate genes obtained by MACE was extended by 5′ RLM-RACE. A list of the gene-specific primers used for the RACE can be found in **Supplementary Table 4**. Based on the MACE and RACE sequences, primers were designed (**Supplementary Table 5**) for amplification and sequencing of the Bale gDNA sequences of the candidate genes. The cDNA and gDNA sequences were aligned to identify introns and exons within the genes. Introns and exons of *INORGANIC PHOSPHATE TRANSPORTER 1* (*BcPHT1*) were identified based on the annotated gDNA sequence from *B. rapa* instead. Then, two gRNAs were designed in an exon near the 5′ end of each gene with a distance of (bp) 69 (*BcFLA1*), 34 (*RAPID ALKALINIZATION FACTOR-LIKE 1*, *BcRALFL1*), 56 (*BcPHT1*), 26 (*ROP GUANINE NUCLEOTIDE EXCHANGE FACTOR 1*, *BcROPGEF1*), 43 (*BcLRL3L1*) and 66 (*BcLRL3L2*), and cloned into pB-CRISPR+35S::GFP. The primers used additionally for the construct preparation for the different genes are shown in **Supplementary Table 6**.

#### Hairy Root Transformation

Constructs were introduced into *B. carinata* cv. Bale by application of the hairy root technique according to Kirchner et al. (2017). In short, constructs were introduced into *Agrobacterium tumefaciens* C58 (ARqua1). Then, the bacterial solution was injected into the hypocotyl of 3-day-old Bale plants, thus inducing hairy roots with an active CRISPR/Cas9 construct.

#### Verification of the Gene Editing

Gene editing of *BcFLA1* was verified by the amplification of the potentially edited gene region (T_a_ = 60°C) and its separation by agarose gel electrophoresis (primers ‘TTCAAACGCAATGGCAACCA’ and ‘ACCCAAATCAAACGACGAGTTTC’). The PCR for it was performed with the KAPA Hifi HotStart PCR Kit (KAPA Biosystems, Wilmington, MA, United States) together with the GC buffer supplied and a primer concentration of 0.3 μM, according to the manufacturer’s instructions. Resulting products were purified from the agarose gel and sequenced. Additionally, indels were detected by polyacrylamide gel electrophoresis (PAGE) (primers ‘GTAAAACGACGGCCAGTTTCAAACGCAATGGCAACCA’ and ‘TTCCGACTCGTGTTGTGTTT’), as described in Kirchner et al. (2017). The primers were designed to bind two different alleles of *BcFLA1* (*BcFLA1a* and *BcFLA1b*), whereby there was one mismatch for *BcFLA1b* in the reverse primer used for the validation by PAGE (base position 9).

### Genomic DNA Isolation

The genomic DNA (gDNA) was isolated using a modified method from Oppermann (2004). All centrifugation steps were performed at 15000 U min^-1^ (4°C). Frozen samples were homogenized similarly to the RNA isolation and resuspended in 1 mL buffer, containing 0.2 M Tris-HCl (pH 9), 0.4 M LiCl, 25 mM EDTA and 1 % (w/v) SDS. After the addition of 50 μL RNase A (10 mg mL^-1^), samples were incubated for 10 min at 60°C, followed by a centrifugation step for 10 min. An amount of 1 mL of the supernatant was mixed for 10 s with 900 mL phenol/chlorophorm/isoamyl alcohol mix (25:24:1) (Roth, Karlsruhe, Germany). Samples were cooled on ice for 5–10 min and centrifuged for 10 min. An amount of 900 μL of the supernatant were mixed for 10 s with 1 mL phenol mix (Roth, Karlsruhe, Germany) and centrifuged for 5 min. Then, 900 μL of the supernatant was put into 1 mL cold isopropanol and the resulting precipitate was pelleted by a centrifugation step of 10 min. The pellet was washed in 1 mL 80% (v/v) EtOH by a centrifugation of 20 min. After drying, the pellet was resuspended in 50 μL TE buffer (pH 9), containing 10 mM Tris and 1 mM EDTA. The gDNA quality and quantity was tested, as described for RNA.

The gDNA sequences were submitted to the NCBI database under the accession numbers listed in **Supplementary Table 2**.

## Results

### Characterization of the Plant Material Used for MACE Regarding the P Deficiency and Root Hair Response

**FIGURE 1:**
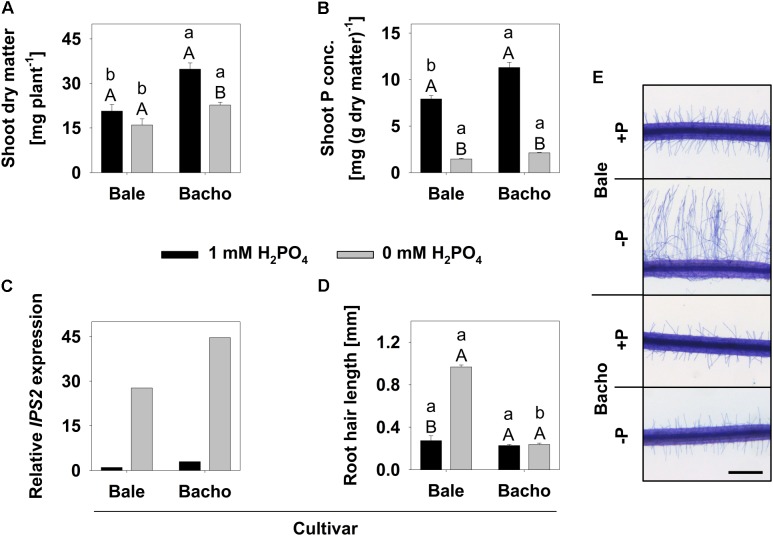
Shoot dry matter (dm) **(A)**, shoot P concentration **(B)**, *AtIPS2* expression **(C)**, root hair length **(D)** and representative root hairs (**E**, scale = 0.5 mm) of *B. carinata* cultivar (cv.) Bale and cv. Bacho as affected by P supply in the 1st experiment (MACE experiment). Small letters denote significant differences at *P* < 0.05 between cv.s at the same P-level; capital letters between *P* treatments of the respective cv. (Tukey test). Columns represent means and bars indicate SE; *n* = 4 **(A,B)** and 8 **(D)** biological replications, whereas 3 technical replications of pooled samples were realized for **(C)**.

Shoot dm, shoot P concentration and expression of a marker gene for P deficiency, *AtIPS2* (Shin et al., 2006), were determined to characterize the P deficiency in the 0 mM H_2_PO_4_ (-P) treatment. The -P treatment in Bacho resulted in a decrease of the shoot dm yield compared to the control (**Figure 1A**). In tendency, this effect also occurred in Bale. However, shoot P concentration in both cv.s was about 2 mg (g dm)^-1^ in the -P treatment and, thus, only one-fifth compared to the shoot P concentration in the 1 mM H_2_PO_4_ (+P) treatment (**Figure 1B**). Furthermore, the *AtIPS2* expression was strongly enhanced during Pi starvation (**Figure 1C**). Bale exhibited a clear increase in the root hair length in the -P treatment compared to the +P treatment, whereas Bacho developed short root hairs under both P conditions (**Figures 1D,E**).

### Analysis of the MACE Data

Between 16 and 26 million tags were produced during the MACE (**Supplementary Figure 1A**). Around four million more tags were obtained in the 0 mM P treatment of both cv.s and six million more tags were produced in both treatments of Bacho compared to Bale. Around 70–80% of the total number of tags could be annotated to *B. rapa* (**Supplementary Figure 1B**), whereas only about 50–60% of the tags could be annotated to *B. napus* (**Supplementary Figure 1C**). About 10% more tags could be annotated in cv. Bacho compared to Bale in both annotations (**Supplementary Figures 1B,C**).

Bale and Bacho strongly differed in the significantly enriched GO terms (**Supplementary Figure 2**). Most of the GO terms significantly enriched in Bale were not significant in Bacho. Especially GO terms related to the root hair development were significantly enriched only in Bale. These categories were root (system) development, cell differentiation, cell wall (organization), Golgi apparatus and cell projection. In the GO categories cell projection, lysosome and negative regulation of cyclin-dependent protein serine/threonine kinase activity the percentage of significantly regulated genes was highest. Mostly more genes were downregulated than upregulated within the GO categories. In Bacho, downregulation was particularly pronounced.

Criteria for the selection of candidate genes with a possible participation in the Pi deficiency-induced root hair elongation were, based on a representative number of tags: (i) significant up- or downregulation under Pi-deficient conditions compared to sufficient conditions in Bale, and (ii) no regulation in Bacho. (iii) Furthermore, the expression level in Bale should be clearly higher (upregulated genes) or lower (downregulated genes) compared to Bacho in the -P treatment. Filtering the dataset according to these criteria resulted in about 200–300 remaining genes in each filter process and annotation (**Supplementary Table 1**), from which 33 candidate genes with a potential regulatory role were selected encoding for arabinogalactan proteins, calcium-related proteins, proteins involved in P transport, protein kinases, GTPase-related proteins, transcription factors, zinc finger proteins, a protein phosphatase, proteins with unknown function and other proteins with a known root hair affecting role (**Supplementary Table 7**). The protein phosphatase was the only selected downregulated gene in Bale under Pi deficiency. Based on the results of the split-root and nutrient specificity experiment, five candidate genes were selected for the functional analysis by knockout and will be further described. Information on all genes is given in **Supplementary Tables 7**–**11**.

**FIGURE 2:**
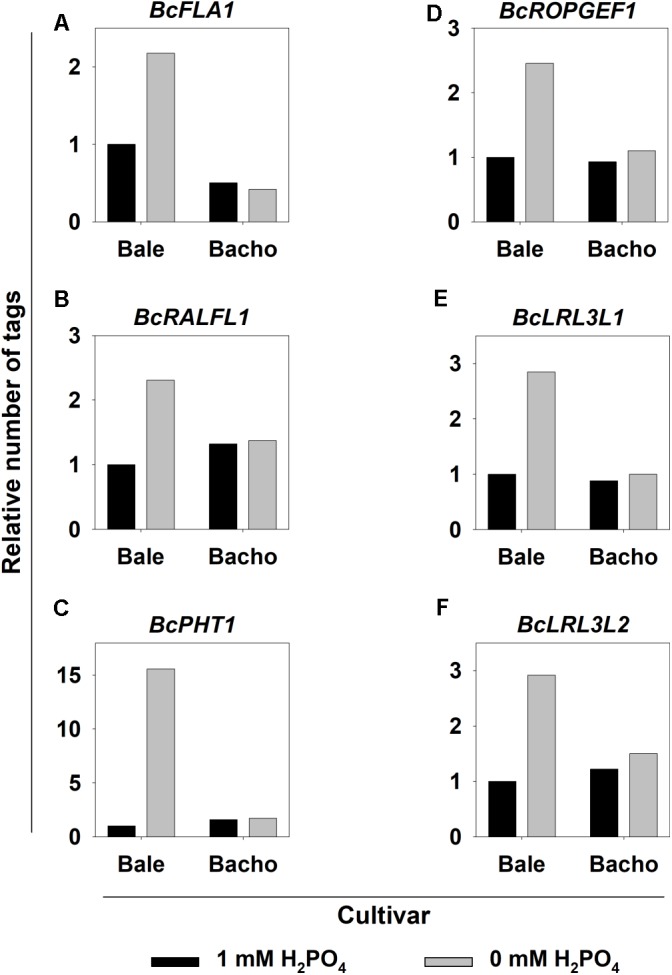
Relative number of normalized tags assigned to the selected candidate genes for increased root hair length under Pi deficiency **(A–F)** in *B. carinata* cv. Bale and cv. Bacho affected by P supply determined by MACE.

The five for the knockout selected candidate genes could be assigned to many of the significantly enriched GO terms, namely root (system) development and cell differentiation (*BcLRL3L*), cell wall organization (*BcROPGEF1*), ion transport and transporter activity (*BcPHT1*), extracellular region (*BcRALFL1*), plasma membrane (*BcFLA1*, *BcPHT1* and *BcROPGEF1*), Golgi apparatus (*BcFLA1*) and cell wall (*BcFLA1* and *BcROPGEF1*, **Supplementary Figure 2**). According to the MACE, most of the candidate genes selected for knockout exhibited a two- to three-times higher expression under deficient P conditions in Bale compared to sufficient P conditions, whereas *BcPHT1* was even 15-fold upregulated (**Figure 2**). The expression of these genes in Bacho was nearly the same under both P conditions.

### Validation of the Candidate Gene Regulation

In the next step, relative gene expression was tested by qPCR in three retained biological replicates originating from the same plant material utilized for the MACE (1st experiment) and in samples obtained from an independently conducted 2nd experiment with an equivalent design to the MACE experiment. For the replicated experiment, P deficiency was confirmed by decreased shoot dm and shoot P concentration (**Supplementary Figures 3A,B**), and root hair length was enhanced in the -P treatment in Bale similarly to the MACE experiment (**Supplementary Figure 3C**).

The up- or downregulated gene expression in Bale under P-deficient conditions was confirmed for 22 and 29 candidate genes in the samples from the MACE (1st) experiment and the 2nd experiment, respectively (**Supplementary Tables 8**, **9**). Overall, the P deficiency effect on the gene regulation in Bale was stronger in the 2nd experiment. Every gene exhibited the expected regulation in Bale in at least one of both experiments. However, *PHOSPHATE TRAFFIC FACILITATOR 1* (*BcPHF1*), *DOMAIN OF UNKNOWN FUNCTION 620* (*BcDUF620*) and *PROTEIN PHOSPHATASE 1* (*BcPP1*) were regulated in Bacho similar to Bale in both experiments, so that they were excluded from the following experiments.

**FIGURE 3:**
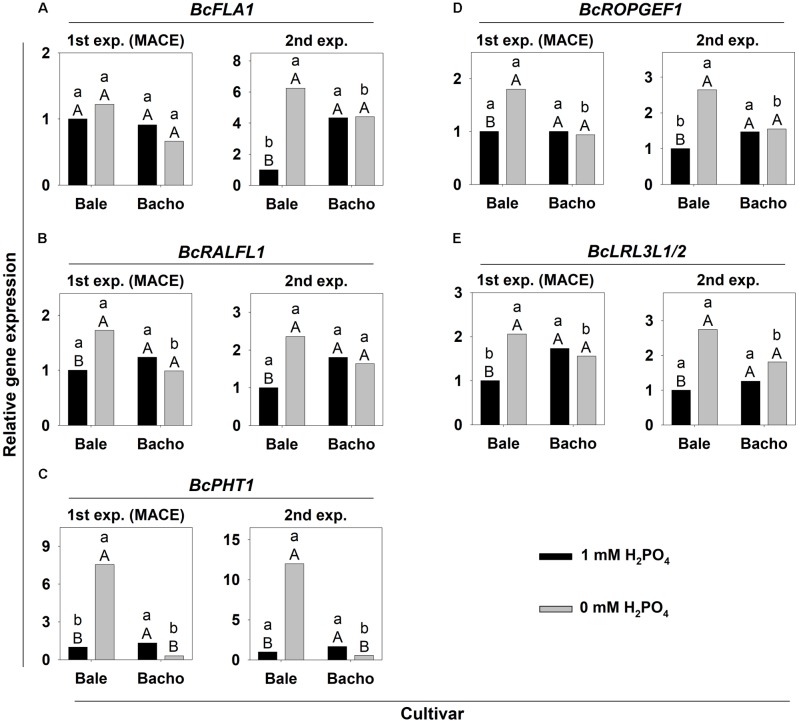
Relative expression of selected candidate genes for increased root hair length under Pi deficiency **(A–E)** in *B. carinata* cv. Bale and cv. Bacho affected by P supply determined by qPCR in samples obtained from the MACE (1st) experiment (exp.) and the independently conducted 2nd exp. *AtUBC9* was used as an endogenous control. Significance was calculated according to Steibel et al. (2009). Small letters denote significant differences at *P* < 0.05 between cv.s at the same P-level; capital letters between P treatments of the respective cv. Columns represent means; *n* = 3.

The upregulation of the five genes which were selected for knockout was confirmed in Bale under Pi deficiency (**Figure 3**). However, the upregulation of *BcFLA1* was significant only in the 2nd experiment (**Figure 3A**). As predicted by MACE, *BcPHT1* exhibited the strongest upregulation and, interestingly, a contrary regulation was observed in Bacho (**Figure 3C**).

### Split-Root Experiment

Bale seedlings were grown in a split-root system in nutrient solutions with (+ +) or without (--) Pi on both sides and with Pi only on one side and without Pi on the other side (+ -) to test whether the candidate genes are involved more in local or systemic signaling pathways.

**FIGURE 4:**
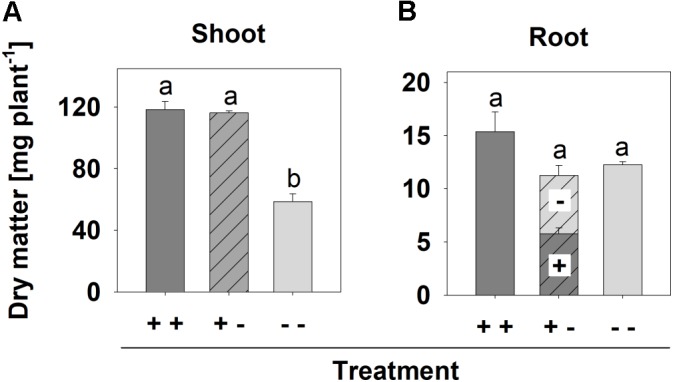
Shoot **(A)** and root **(B)** dm of *B. carinata* cv. Bale affected by P supply in a split-root system. Different letters denote significant differences at *P* < 0.05 (Tukey test). Columns represent means and bars indicate SE; *n* = 3.

After 8 days in the split-root system, shoot growth of the [+ +] and [+ -] treatment was similar (**Supplementary Figures 4A,B**), but was clearly less in the [--] plants, which had small dark green leaves (**Supplementary Figure 4C**), typical P deficiency symptoms. Accordingly, the shoot dm yield of the [--] plants was about 50% lower than the other treatments (**Figure 4A**). However, the root dm yield was not significantly affected by any of the treatments (**Figure 4B**).

**FIGURE 5:**
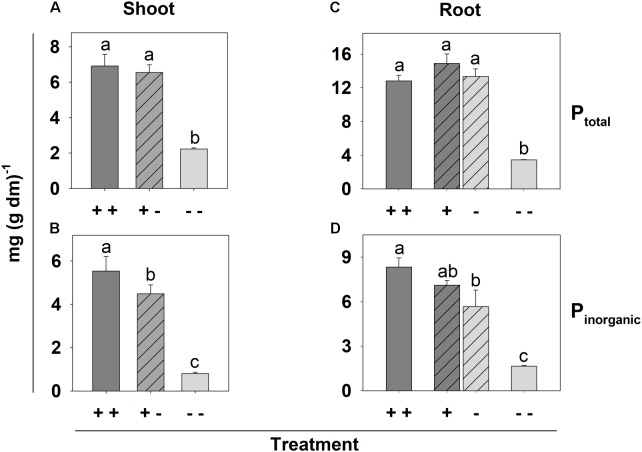
P_total_ concentration in shoot **(A)** and root **(B)** as well as P_inorganic_ concentration in shoot **(C)** and root **(D)** of *B. carinata* cv. Bale affected by P supply in a split-root system. Different letters denote significant differences at *P* < 0.05 (Tukey test). Columns represent means and bars indicate SE; *n* = 3.

The similar shoot dm yield of the [+ -] and [+ +] plants indicated that the overall P status was sufficient in the [+ -] plants, which was in line with a high P_total_ concentration in shoot and root dm in both treatments (**Figures 5A,B**). By contrast, the P_total_ concentration in the shoot and root dm of the [--] plants was reduced to about 1/3. The P_inorganic_ concentration was reduced steepest in shoots and roots of the [--] plants (**Figures 5C,D**). However, the shoots of the [+ -] plants and the related root halves grown on the P deficient side ([-] roots) also exhibited a decrease of the Pi concentration compared to the [+ +] plants.

**FIGURE 6:**
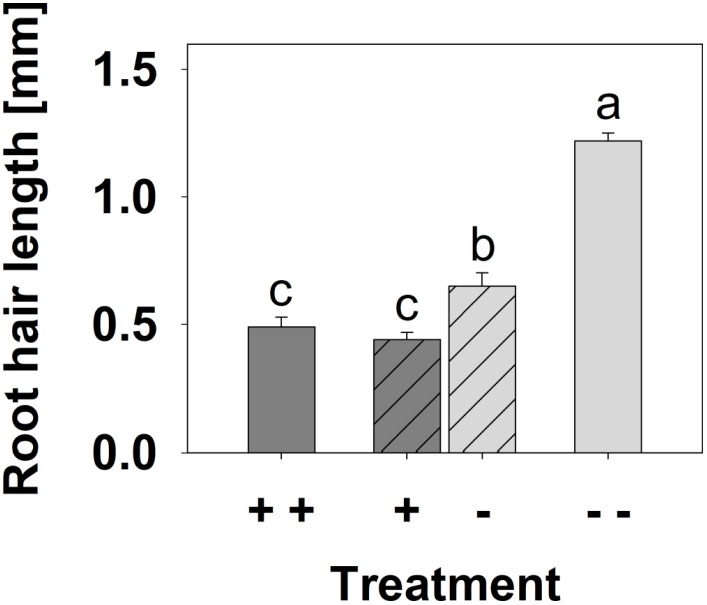
Root hair length of *B. carinata* cv. Bale affected by P supply in a split-root system. Different letters denote significant differences at *P* < 0.05 (Tukey test). Columns represent means and bars indicate SE; *n* = 30.

Root hair length of the [--] plants was enhanced by a factor of 2.5 compared to the [+ +] plants (**Figure 6**), whereas root hair length in the [+ -] treatment was increased by 30 % in the -P side, but not affected in the +P side compared to the [+ +] treatment.

**FIGURE 7:**
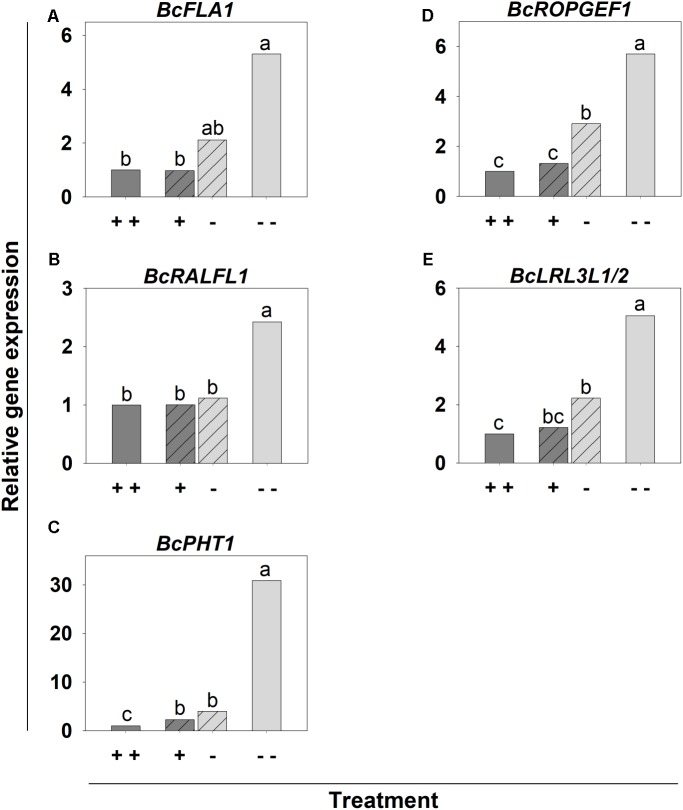
Relative expression of candidate genes for increased root hair length during Pi deficiency **(A–E)** in *B. carinata* cv. Bale affected by P supply in a split-root system. *AtUBC9* was used as an endogenous control. Significance was calculated according to Steibel et al. (2009). Different letters denote significant differences at *P* < 0.05. Columns represent means; *n* = 3.

The upregulation of the candidate genes by Pi deficiency ([--] treatment) was confirmed (**Supplementary Table 10** and **Figure 7**). Twenty-five of the 30 candidate genes in the [+ -] treatment were upregulated in the -P side. However, in most of the cases, this upregulation was not as high as in the [--] treatment. Three candidate genes, namely *ARABINOGALACTAN PROTEIN 2* (*BcAGP2*), *BcRALFL1* and *SHAVEN3-LIKE* (*BcSHV3L*), were not affected by the P supply in the split-root system (**Supplementary Table 10** and **Figure 7B**). Conversely, a few genes, most of all *CALCIUM-BINDING EF-HAND FAMILY PROTEIN 1 (BcCaBP1)* and *EXOCYST COMPLEX COMPONENT EXO70-LIKE* (*BcEXO70L*), were nearly as strongly upregulated in the roots of the -P side as in the [--] treatment (**Supplementary Table 10**). *BcPHT1* exhibited the strongest upregulation in the [--] treatment, whereas the upregulation in the split-root variant was only 2–4-fold in the [+] and [-] side, respectively (**Figure 7C**). The expression level of *BcFLA1*, *BcROPGEF1* and *BcLRL3L1/2* in the [-] side of the [+ -] treatment was higher than in the sufficiently supplied roots, but lower compared to the [--] treatment (**Figures 7A,D,E**).

### Investigation of the Nutrient Specificity of the Candidate Genes

Bale and Bacho were also grown in nutrient solutions lacking N and K, respectively, to check whether the successfully validated candidate genes are regulated specifically during root hair growth under Pi starvation.

**FIGURE 8:**
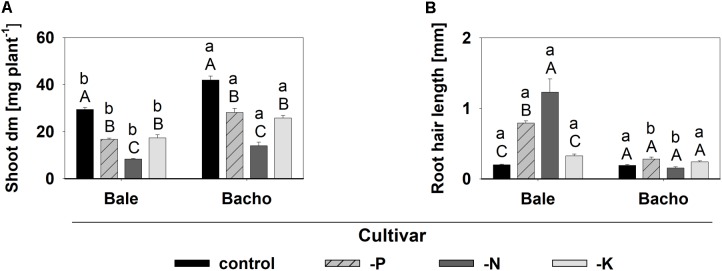
Shoot dm **(A)** and root hair length **(B)** affected by nutrient deficiency. Significant differences at *P* < 0.05 are denoted by small letters between cv.s for the same nutrient treatment and capital letters between nutrient treatments for the same cv. (Tukey test); columns represent means and bars indicate SE; *n* = 6 **(A)** and *n* = 12 **(B)**, respectively.

The lower shoot dm yield (**Figure 8A**) and nutrient concentration in the shoot dm (**Supplementary Figure 5**) in the deficiency variants compared to the control with sufficient nutrient supply indicated deficiency for all three nutrients in both Bale and Bacho. Root hair length was increased under P and N deficiency in Bale, but not in Bacho (**Figure 8B** and **Supplementary Figure 6**) as was expected from previous studies (Bremer, 2010). The N deficiency even led to a higher root hair length than P deficiency in Bale. The K deficiency did not affect the root hair length in either cv.

**FIGURE 9:**
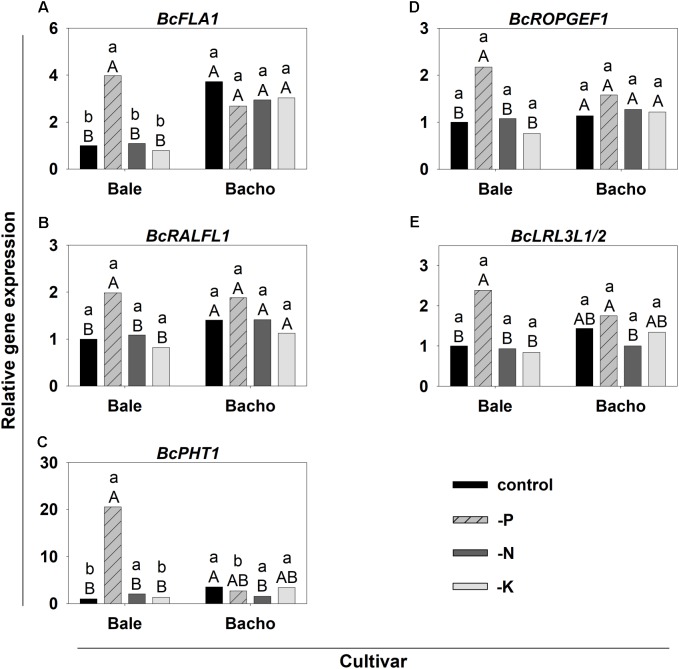
Relative expression of candidate genes for increased root hair length during Pi deficiency **(A–E)** in *B. carinata* cv. Bale and cv. Bacho affected by nutrient deficiency determined by qPCR. *AtUBC9* and *BcEF-1-a1* were used as endogenous controls. Significance was calculated according to Steibel et al. (2009). Significant differences at *P* < 0.05 are denoted by small letters between cv.s for the same nutrient treatment and capital letters between nutrient treatments for the same cv. Columns represent means; *n* = 3.

Most of the 30 candidate genes for Pi deficiency-induced root hair growth were upregulated in Bale by both Pi and N deficiency compared to sufficient conditions (**Supplementary Table 11** and **Figure 9**). However, the increase of the expression in most of the cases was at least higher in tendency under Pi deficiency compared to N deficiency. Interestingly, four genes (*BcFLA1*, *BcRALFL1*, *BcROPGEF1*, and *BcLRL3L1/2*) were specifically upregulated by Pi deficiency (**Figures 9A,B,D,E**) and the upregulation of *BcPHT1* under Pi deficiency was many times over that during N deficiency (**Figure 9C**). By contrast, K deficiency did not affect the expression of the candidate genes. No significant upregulation in Bacho was observed for any genes and nutrient deficiencies compared to sufficient conditions, except for an upregulation of the expression of *CYCLIC NUCLEOTIDE-GATED CATION CHANNEL BETA-1-LIKE* (*CNGB1L*) under N deficiency.

### Knockout of Candidate Genes by CRISPR/Cas9

According to the prior experiments, five candidate genes were selected for further functional investigation by knockout using CRISPR/Cas9. These included the four P-specific reacting genes *BcFLA1*, *BcRALFL1*, *BcROPGEF1* and *BcLRL3L1/2* (**Figures 9A,B,D,E**). Furthermore, *BcPHT1* was selected because of its very strong upregulation under Pi deficiency in Bale, while it was downregulated in Bacho (**Figure 3C**).

**FIGURE 10:**
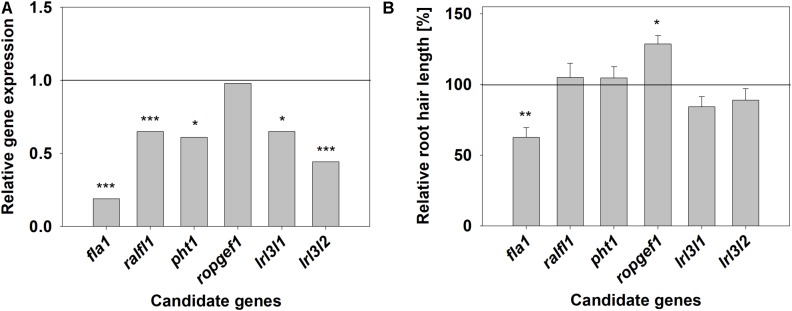
Relative candidate gene expression **(A)** and relative root hair length of the transgenic hairy roots **(B)** after targeting the respective candidate genes for increased root hair length under Pi deficiency by CRISPR/Cas9. Horizontal lines represent the average wildtype expression and root hair length, respectively. Stars denote significant differences, determined according to Steibel et al. (2009) **(A)** and Tukey test **(B)** (significance codes: 0 ‘^∗∗∗^’ 0.001 ‘^∗∗^’ 0.01 ‘^∗^’ 0.05). Columns represent means for *n* = 4–8 **(A)** and 5–14 **(B)**, respectively. Bars in **(B)** indicate SE.

The gene expression of all candidate genes, except for *BcROPGEF1*, was reduced in the transgenic CRISPR roots compared to the control (**Figure 10A**). Expression of *BcFLA1* was reduced to 20% and *BcLRL3L2* expression to 45%. *BcRALFL1*, *BcPHT1* and *BcLRL3L1* expression was reduced to about 60%. The reduction in case of *BcFLA1* (**Supplementary Figure 7A**), *BcRALFL1* (**Supplementary Figure 7B**) and both *BcLRL3L* isoforms (**Supplementary Figures 7E,F**) was relatively homogenous in the different transgenic roots, whereas the expression of the other genes exhibited strong fluctuations (**Supplementary Figures 7C,D**).

The root hair length was reduced only in the *bcfla1* roots (to about 60% compared to the control) (**Figure 10B** and **Supplementary Figure 8**). By contrast, the *bcropgef1* roots exhibited about 30% longer root hairs.

**FIGURE 11:**
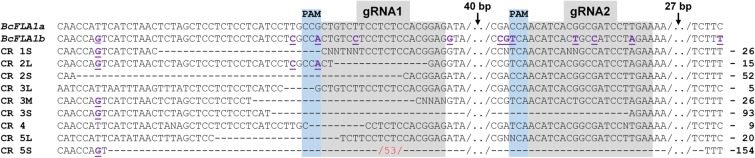
CRISPR/Cas9-induced deletions and insertions in *BcFLA1*. gRNA regions (gray), PAM sequence (blue), number of bp inserted (red) and number of bp deleted (on the right). Numbers on the left indicate the respective transgenic roots, whereby ‘L’ indicates a long fragment (small deletion), ‘M’ a medium fragment and ‘S’ a small fragment (large deletion). Nucleotides used for differentiation of *BcFLA1b* from *BcFLA1a* (violet, underlined). CR = CRISPR, /../ = break.

Since the strong downregulation of *BcFLA1* was accompanied by a reduction in the root hair length, we took a closer look at the gene-editing events caused by CRISPR/Cas9. Sanger sequencing detected deletions in *BcFLA1* in all replications ranging from 5 to 154 bp (**Figure 11**). Both alleles of *BcFLA1* were affected. Up to three different deletions were observed per replication (transgenic root). However, only two deletions (CR 3S and CR 5S) spanned both gRNA regions. All other deletions occurred exclusively in the gRNA1 region. Furthermore, a 53° bp insertion occurred in CR 5S, the replication with the largest deletion of 154 bp. There was often an overlapping of at least two sequences in the sequencing chromatograms starting at gRNA1, which indicates that several amplicons with different gene-editing events were purified at once.

**FIGURE 12:**
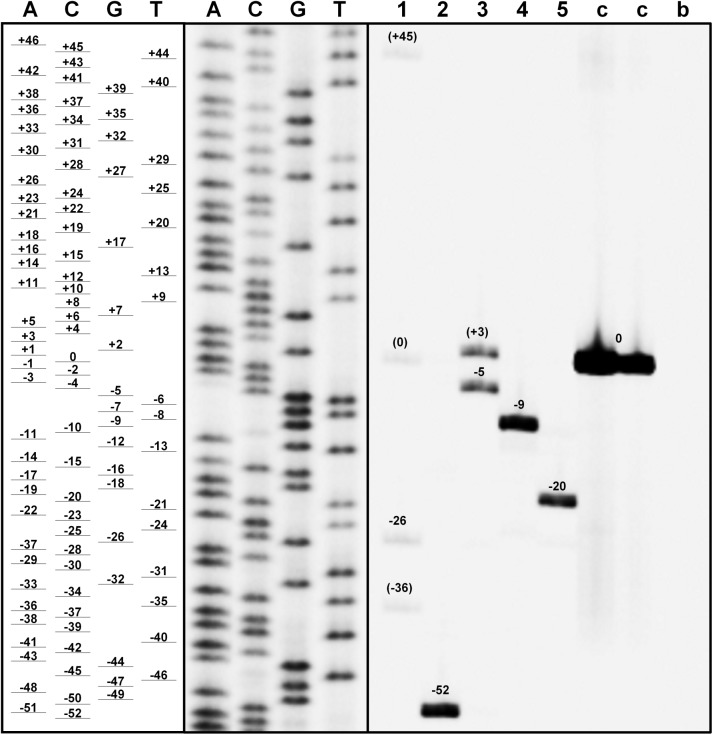
Validation of the CRISPR/Cas9-induced gene-editing of *BcFLA1* by fragment analysis by PAGE. A, C, G and T = Sanger sequencing reactions with the corresponding dideoxynucleotides; c, control; b, blank (no template control). Lane numbers indicate the respective transgenic roots. Numbers close to the bands indicate the bp deleted (–) and inserted (+) compared to the wildtype size (0). Brackets indicate indels with approximated size, which did not occur during sequencing.

The fragment analysis by PAGE uncovered indels ranging from -52 to +45 bp (**Figure 12**). Three replications (2, 4, and 5) exhibited only one gene-editing event, whereas one insertion and one deletion occurred in replication 3. Replication 1 even exhibited two deletions and one insertion, whereby there was some of the wildtype sequence left. The intensity of the amplicon bands and therefore, the frequency of the gene-editing events was similar within the replications.

**FIGURE 13:**
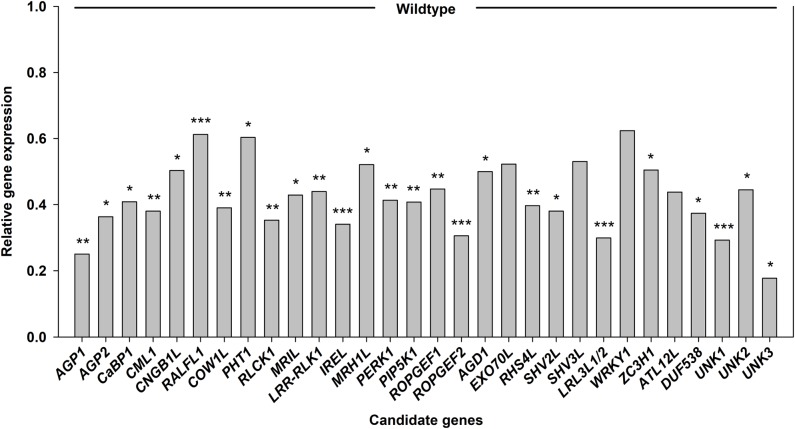
Relative expression of candidate genes for Pi deficiency-induced root hair growth in *bcfla1* roots grown under Pi-deficient conditions. *AtUBC9* was used as an endogenous control. Significance was calculated according to Steibel et al. (2009) (significance codes: 0 ‘^∗∗∗^’ 0.001 ‘^∗∗^’ 0.01 ‘^∗^’ 0.05). Columns represent means; *n* = 3.

The expression of the other candidate genes was analyzed in the *bcfla1* roots to investigate where *BcFLA1* may be working in the signal transduction cascade leading to the Pi deficiency-induced root hair elongation. Most of them exhibited a clear decrease in their expression (**Figure 13**), whereby the deepest reduction occurred for *UNKNOWN 3* (*BcUNK3*) (to a level below 20%).

## Discussion

### Identification of Regulatory Candidate Genes for Pi Deficiency-Induced Root Hair Elongation

The aim of the present study was to identify candidate genes for the Pi deficiency-induced root hair elongation in *B. carinata*. Consequently, we used two different cv. of *B. carinata* differing in their root hair length under Pi starvation. cv. Bale develops long root hairs in response to Pi deficiency, whereas cv. Bacho develops short root hairs under both sufficient and deficient Pi conditions (Eticha and Schenk, 2001). Genome-wide expression profiles of Bale and Bacho under low (0 mM) and high (1 mM) Pi conditions were received by MACE. Therefore, RNA was extracted from 2-cm root tips, because this length ensures the receipt of root hairs with different developmental stages from the initiation to the termination of the root hair growth, so that the genes participating in the root hair elongation should also be expressed in the harvested plant material. The reduced shoot dm and shoot P concentration in both Bale and Bacho and the upregulation of the marker gene expression (**Figures 1A–C**) confirmed P starvation in the respective treatment of the MACE experiment. Additionally, longer root hairs were induced exclusively in Bale and not in Bacho under Pi deficiency (**Figure 1D**), as was expected from preliminary studies (Eticha and Schenk, 2001; Bremer, 2010), so that the plant material was defined as suitable for the subsequent MACE.

Since no database was available for *B. carinata*, the tags produced in the MACE were annotated to *B. rapa* and *B. napus*, which are, as *B. carinata*, a member of the Triangle of U (Li et al., 2017). The latter predicts a higher similarity between *B. carinata* and *B. napus*. However, more tags could be annotated to *B. rapa* than *B. napus* (**Supplementary Figure 1**). This may be due to the different annotation methods and the degree of completeness of the databases. Nevertheless, we used both annotations to have a greater basis for the candidate gene selection.

According to the root hair response, we selected genes exhibiting a clear down- or upregulation under Pi deficiency in Bale and no regulation in Bacho. Furthermore, only genes with a potential regulatory function were chosen, because we were interested in genes regulating the root hair elongation according to the Pi availability rather than genes involved in the general root hair elongation process. According to their function or structure, the 33 candidate genes were assigned to nine groups. Finally, five candidate genes were selected for functional analysis by knockout: (i) Since we were most interested in regulatory genes transmitting the signal of low P to the general root hair elongation pathway, we chose the four P-specific reacting candidate genes *BcFLA1*, *BcRALFL1*, *BcROPGEF1* and *BcLRL3L1/2* (**Figures 9A,B,D,E**). (ii) *BcPHT1* was selected because of its very strong upregulation under Pi deficiency in Bale and the contrary regulation in Bacho (**Figure 3C**), which may indicate a role in P-sensing analogous to nitrate-sensing by nitrate transporter NRT1.1 (Krouk et al., 2010). A more detailed description of these genes can be found in **Supplementary Text 1**.

There was a good overall agreement between the regulation of the candidate genes observed in the MACE and in the qPCR (**Supplementary Tables 7**, **8**). Even stronger upregulations were observed in the 2nd experiment, so that it actually correlated better to the MACE than the biological replications from the MACE (1st) experiment itself (**Figure 3** and **Supplementary Table 9**). Furthermore, the regulation of the candidate genes was confirmed in the split-root and nutrient specificity experiments (**Supplementary Tables 10**, **11**).

In addition to the candidate genes which were selected after filtering the MACE data, *BcPIP5K1* was investigated, because its counterpart in *A. thaliana* (*AtPIP5K3*) was predicted to have a key role in increasing the root hair elongation response to Pi starvation, at least at young seedling stages. However, in this study, *BcPIP5K1* was upregulated by Pi deficiency both in Bale and Bacho (**Supplementary Table 8**), indicating that it has another or an additional role in *B. carinata* or, at least, in Bacho.

### Investigation of the Candidate Gene Characteristics Regarding Their Role in Signaling and Nutrient Specificity

Genes, whose regulation according to MACE was confirmed in at least one of the two independent experiments, were further investigated regarding their role in signaling and nutrient specificity.

Bale seedlings were grown in a split-root system containing sufficient (+ +) or deficient (--) Pi on both sides or sufficient Pi only on one and deficient Pi on the other side (+ -) to investigate whether the effect of Pi deficiency on root hair growth in *B. carinata* cv. Bale is mediated locally or systemically. Reduced shoot dm yield and low shoot P_total_ conc. indicated strong Pi deficiency in the [--] treatment (**Figures 4A**, **5A**), accompanied by clear Pi deficiency symptoms on these plants (**Supplementary Figure 4**). However, shoot dm yield, P_total_ concentration and phenotype in the [+ -] treatment were similar to the [+ +] treatment, indicating that the Pi uptake on the +P side was enough for an overall sufficient P status of the plants. In addition to the P_total_ concentrations, we measured the P_inorganic_ concentrations in the shoots and roots, because Pi itself could serve as signaling molecule in transmitting the low Pi signal (Chiou and Lin, 2011). In contrast to P_total_, P_inorganic_ also decreased in the shoots and in the -P side roots of the [+ -] treatment compared to the [+ +] treatment (**Figures 5C,D**). P_inorganic_ represents the storage form contained in the vacuoles (Bieleski, 1973). In this storage pool, a lower P supply in the [+ -] treatment compared to the [+ +] treatment was reflected. However, the root halves of the [+ -] treatment were not different, suggesting that the slightly enhanced root hair length (**Figure 6**) and increased gene expression in the -P side roots of this treatment were not caused by P_inorganic_, but local Pi deficiency in the medium. This fits with the results of Thibaud et al. (2010), who concluded that local P deficiency responses are mediated by the external Pi. However, systemic signaling also seems to have an influence, because the root hair length of the roots from the -P side was not as strongly increased as in the [- -] treatment (**Figure 6**). The assumption that both local and systemic signaling play a role in the Pi deficiency-induced root hair elongation was supported by the expression level of most of the candidate genes in the root tips from the -P side of the split-root variant, which was between the level in the root tips grown in the sufficient P solutions and that of the [--] treatment (**Supplementary Table 10**). However, three candidate genes, among them *BcRALFL1* (**Figure 7B**), did not exhibit any local P response in the split-root variant, so they may be involved exclusively in systemic signaling pathways. Alternatively, two genes (*BcCaBP1* and *BcEXO70L*) exhibited a strong local P deficiency response, which was nearly as high as in the [--] treatment, thus, they may particularly play a role in local signaling pathways (**Supplementary Table 10**). Therefore, both local and systemic signaling may play a role in the Pi deficiency-induced root hair elongation in *B. carinata*.

Bale and Bacho were cultivated additionally under N- and K deficiency to uncover candidate genes which are specifically reacting in response to Pi deficiency. After validation of the nutrient deficiencies by a reduced shoot dm (**Figure 8A**) and nutrient concentration (**Supplementary Figure 5**), the expression of the candidate genes was determined (**Supplementary Table 11**). Interestingly, none of the candidate genes reacted in response to K deficiency, which was the only deficiency that did not lead to an enhanced root hair length (**Figure 8B**). This strongly indicates that the candidate genes are indeed directly or indirectly involved in the root hair elongation and not in general nutrient deficiency pathways. However, most of the candidate genes were upregulated by both P and N deficiency in Bale (**Supplementary Table 11**), which indicates that these genes are more involved in the general root hair elongation pathway. By contrast, the four candidate genes specifically upregulated under Pi deficiency: *BcFLA1*, *BcRALFL1*, *BcROPGEF1* and *BcLRL3L1/2* (**Figures 9A,B,D,E**) may play a role in the regulation of the root hair elongation pathway dependent on the Pi status. The FLAs have not been associated with Pi deficiency so far. However, it has been shown in several studies that they have the ability to counteract abiotic stress by altering gene expression (Zang et al., 2015).

### Functional Characterization of the Candidate Genes by Knockout via CRISPR/Cas9

The most promising candidate genes were targeted by CRISPR/Cas9 to cause a knockout of the gene function and the effects on the root hair length under Pi deficiency were investigated. Out of the targeted genes, downregulation of *BcFLA1* led to a significantly reduced root hair length (**Figure 10**), which is a strong evidence for a role of *BcFLA1* in the Pi deficiency-induced root hair elongation in *B. carinata*.

Since the number of transcripts derived from a functional gene may be overestimated by qPCR, because of an ineffective nonsense-mediated mRNA decay in the case of an absent frameshift mutation, we further investigated the CRISPR/Cas9-induced gene-editing events in *BcFLA1* by Sanger sequencing and fragment analysis by PAGE. In most of the cases, relatively large deletions occurred in both alleles of *BcFLA1*, indicating that the knockout of the gene function was successful (**Figure 11**). However, not all gene editing events could be separated by agarose gel electrophoresis. Therefore, a PAGE was performed to separate the amplicons of the potentially edited gene region with a higher resolution and to estimate their frequency. Indeed, if the loading of the gel was sufficiently high, some additional bands became visible on the polyacrylamide gel compared to Sanger sequencing (**Figure 12**). Hence, we were able to separate all amplicons and determine the indel sizes more precisely. Furthermore, the intensity of the amplicon bands allowed the estimation of the quantity of every indel. Even if the bands of the first replication were weak due to a low amount of DNA, four bands of similar intensity were observed from which one may represent the wildtype sequence. By contrast, the wildtype sequence in the other replications was completely absent. Some of the indels may not have induced a frameshift mutation, but it can be assumed that the gene function was knocked out due to the large size of most of the indels. This fits with the strong and relatively homogenous downregulation of the *BcFLA1* gene expression measured by qPCR (**Figure 10A** and **Supplementary Figure 7A**), since a possible self-stimulation of the *BcFLA1* expression may be impeded due to a defective BcFLA1 protein. Additionally, as the small indels around gRNA1 affected the N-terminal signal peptide of BcFLA1 (**Supplementary Table 12**), this may have caused a mislocalization of BcFLA1, so that it could not perform its normal function in the cell wall and/or the extracellular matrix.

The decreased transcript level of BcFLA1 was accompanied by a reduced root hair length of the *bcfla1* roots (**Figure 10B** and **Supplementary Figure 8A**). However, even if the specificity of the CRISPR/Cas9 mechanism is relatively high in plants (Feng et al., 2014; Zhang et al., 2014; Jacobs et al., 2015), off-target effects cannot be excluded, especially if non-model plants without the full genome information are used, as in our study. Therefore, we developed a complementation variant with an additional overexpressed version of *BcFLA1*, which was resistant to the CRISPR/Cas9 mechanism in combination with the two gRNAs used in this study (for details, see Kirchner et al., 2017). The complementation of the *BcFLA1* gene function led to a significant increase of the root hair length compared to the CRISPR variant, thus, it was confirmed that the shorter root hairs of the transgenic CRISPR roots arose from the downregulation of *BcFLA1*.

In the last step, we wanted to test whether the downregulation of *BcFLA1* had an influence on the expression of the other candidate genes. Unexpectedly, nearly all candidate genes were strongly downregulated in the *bcfla1* root tips (**Figure 13**). This may indicate that *BcFLA1* indeed has a role at the beginning of the signal transduction cascade leading to a higher root hair length under Pi deficiency. However, this result can be falsified, because the number of root hairs may also be affected in *bcfla1*. A lower number of root hairs would automatically lead to an overall reduced expression of root hair-related genes in the sample. Therefore, the number of root hairs should be taken into account in further analyses to confirm this result.

Considering all results, we suggest a regulating role for *BcFLA1* in the Pi deficiency-induced root hair elongation. The specific upregulation of *BcFLA1* in response to Pi deficiency and the effect of its altered expression on the root hair length indicates that *BcFLA1* acts in a P-specific pathway which may translocate the low Pi signal to the root hair elongation pathway. This P-specific pathway may merge with a pathway translocating environmental cues to a putative root hair-specific transcription factor which senses both developmental and environmental signals and controls downstream root hair genes, thus, leading to a balanced root hair elongation (Hwang et al., 2016).

**FIGURE 14:**
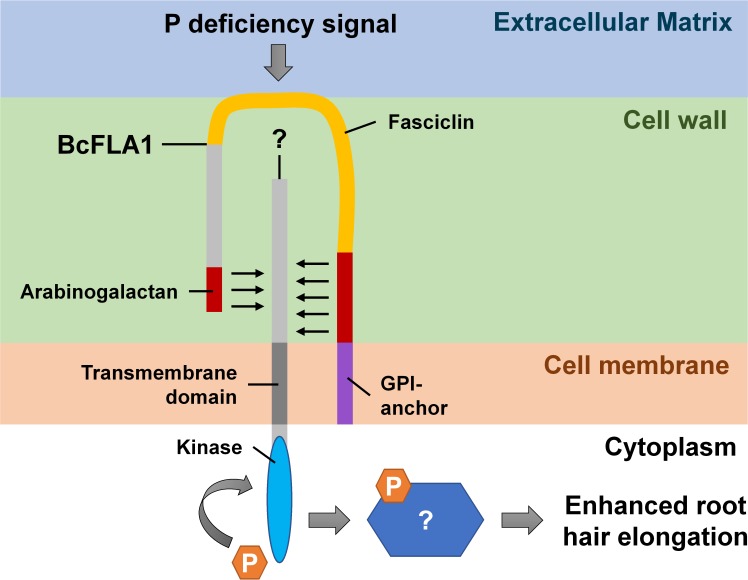
Model of the potential position and working mechanism of BcFLA1.

Another open question is the functional mechanism of the putative signal perception and transduction by BcFLA1. The FLAs can have a key function in transmitting environmental changes to the cell, as they connect the cell with neighboring cells or the extracellular matrix physically (Faik et al., 2006). Via their fasciclin (FAS) domains, they may mediate protein-protein interactions by facilitating the adhesion of plasma membrane or cell wall-associated ligands (Tan et al., 2012). A topology prediction of BcFLA1 revealed that the C-terminal end of BcFLA1 is embedded in the plasma membrane via a GPI-anchor and that the rest of the protein is outside the cell (**Figure 14**). Since the protein consists of two AG-rich regions surrounding the FAS domain (**Supplementary Table 12**), it could be speculated that the first AG-rich region of BcFLA1 passes through the cell wall, the FAS domain is located on the surface of the cell wall with contact to the extracellular matrix and the second AG-rich region is again located in the cell wall (**Figure 14**). Low Pi signal perception could, therefore, be mediated by the interaction of the FAS domain with signal molecules in the extracellular matrix. The signal may then be transmitted to a putative cell wall protein kinase, whose extracellular domain may be connected to the AG regions of BcFLA1 within the cell wall, which was proposed in a model regarding another FLA protein in Arabidopsis (Basu et al., 2016). This may, in turn, lead to autophosphorylation of the cytosolic kinase domain of the putative protein kinase, leading to downstream signaling events for the Pi deficiency-induced root hair elongation. Furthermore, the GPI anchor could be cleaved from BcFLA1 so that it is released from the plasma membrane, possibly leading to further signaling events. Interestingly, a role in elongation processes was documented for several FLAs, for example, the involvement of GhFLA1 in the initiation and elongation of cotton fibers (Huang et al., 2013) and AtFLA4, which maintains cell expansion during salt stress (Tan et al., 2012). Furthermore, another FLA was preferentially expressed in root hairs of Arabidopsis and was upregulated at the protein level in response to Pi starvation (Salazar-Henao and Schmidt, 2016), which may indicate the involvement of several FLAs in the Pi deficiency-induced root hair growth.

Taken together, the results of this study in combination with the proposed functional abilities of fasciclin-like AGPs strongly indicate a role of *BcFLA1* in the Pi deficiency-induced root hair elongation.

## Author Contributions

TK and MS planned the experiments. TK, MN, KR, and TL conducted the experiments. MH provided the CRISPR vector. TK performed the data analysis, visualization, and writing the manuscript. MS and HK supervised and revised the study.

## Conflict of Interest Statement

The authors declare that the research was conducted in the absence of any commercial or financial relationships that could be construed as a potential conflict of interest.
