# Supplementary material for: Molecular Background of Pi Deficiency-Induced Root Hair Growth in Brassica carinata – A Fasciclin-Like Arabinogalactan Protein Is Involved
Source: Front Plant Sci. 2018 Sep 19;9:1372. doi: 10.3389/fpls.2018.01372 (PMC6157447; doi:10.3389/fpls.2018.01372)
Supplement: Supplementary file 1 [file Data_Sheet_1.docx]

Supplementary Material

Molecular Background of Pi Deficiency-Induced Root Hair Growth in *Brassica carinata* – a Fasciclin-Like Arabinogalactan Protein Is Involved

**Thomas W. Kirchner, Markus Niehaus, Kim L. Rössig, Timo Lauterbach, Marco Herde, Manfred K. Schenk^*^**

*** Correspondence:** Corresponding Author: schenk@pflern.uni-hannover.de

# Supplementary Figures


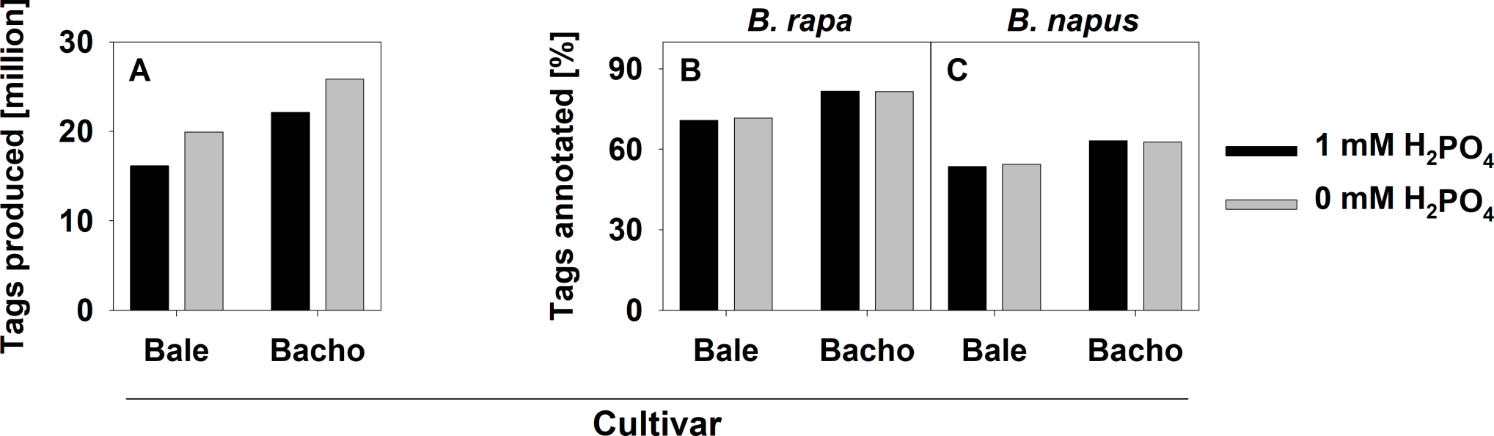


**Supplementary Figure 1.** Tags produced during MACE in millions (A) and relative number of tags annotated to *Brassica rapa* (B) and *Brassica napus* (C).


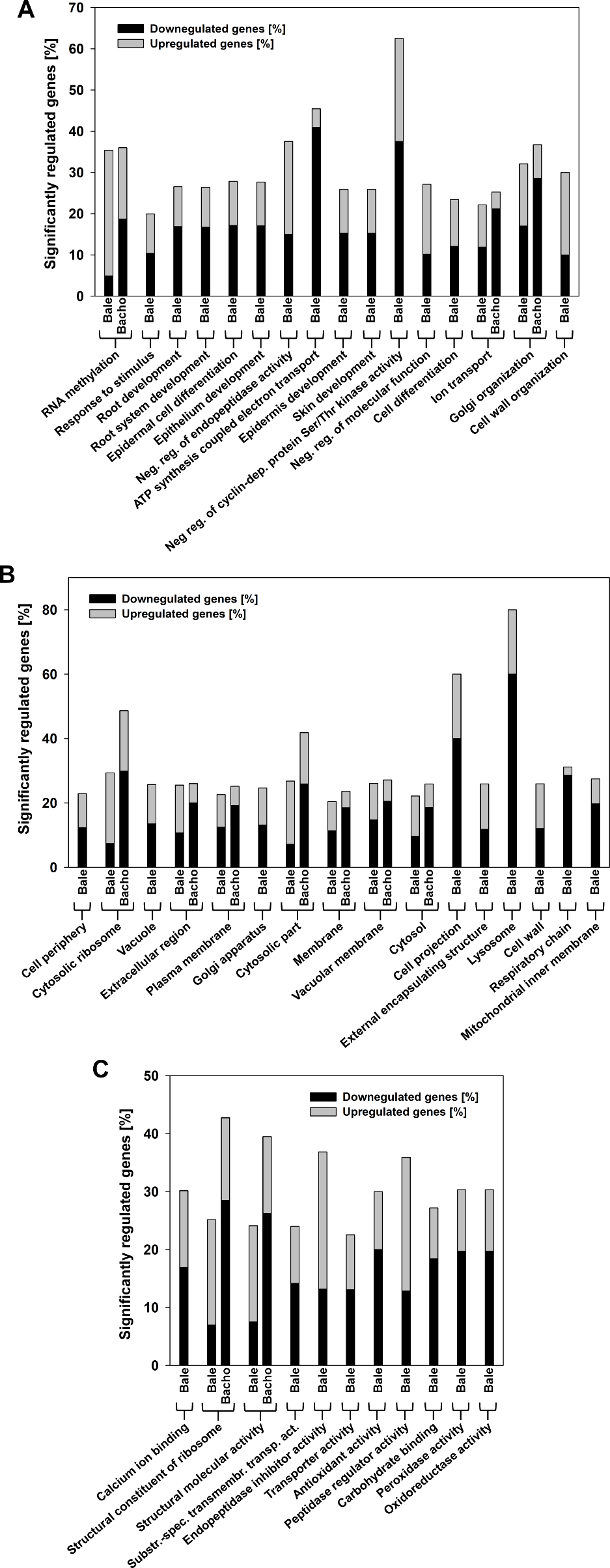


**Supplementary Figure 2.** Significantly regulated genes in significantly enriched GO terms in Bale and Bacho. Displayed are all significantly enriched GO terms in Bale (p<0.01). If no data was shown for Bacho, the enrichment for the corresponding GO term was not significant. The GO categories were sorted from left to right according to significance with increasing p-value. Biological process (A), cellular component (B) and molecular function (C).


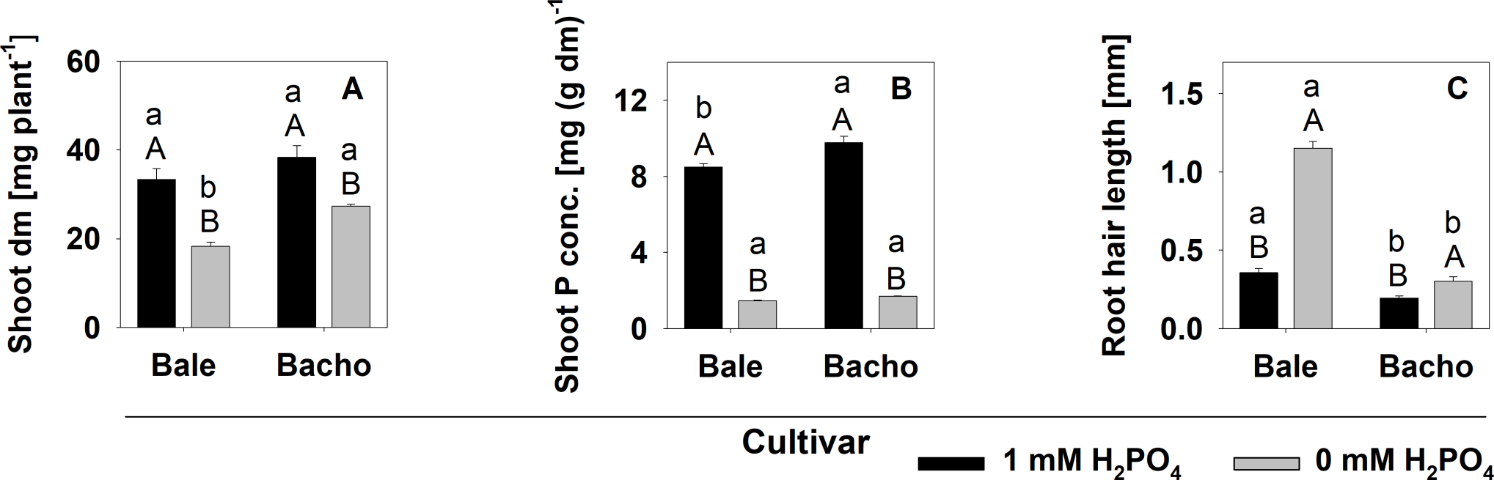


**Supplementary Figure 3.** Shoot dry matter (dm) (A), shoot P concentration (B) and root hair length (C) of *B. carinata* cv. Bale and cv. Bacho affected by P supply in the 2^nd^ experiment (exp.). Small letters denote significant differences at P < 0.05 between cv.s at the same P-level; capital letters between P treatments of the respective cultivar (Tukey test). Columns represent means and bars SE; n = 6 (A), 3 (B) and 12 (C).


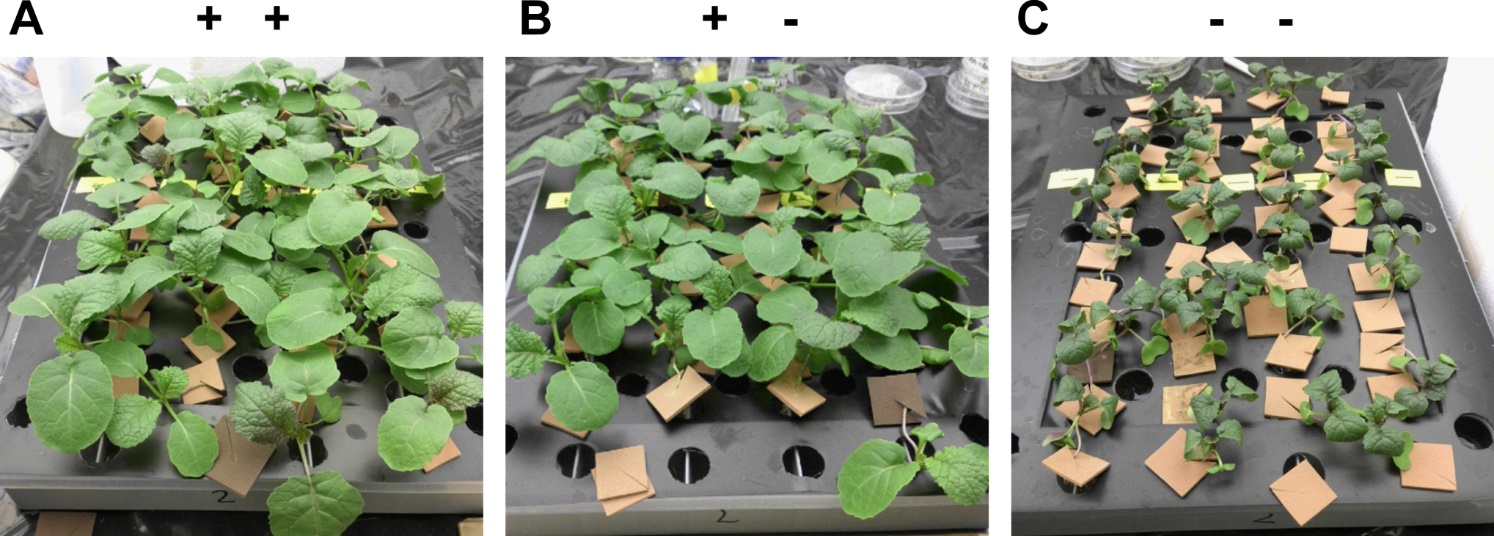


**Supplementary Figure 4.** *B. carinata* cv. Bale plants after an eight-day cultivation in a split-root system containing 1 mM Pi on both sides (A), 1 mM Pi on one side and 0 mM Pi on the other side (B), and 0 mM Pi on both sides (C).


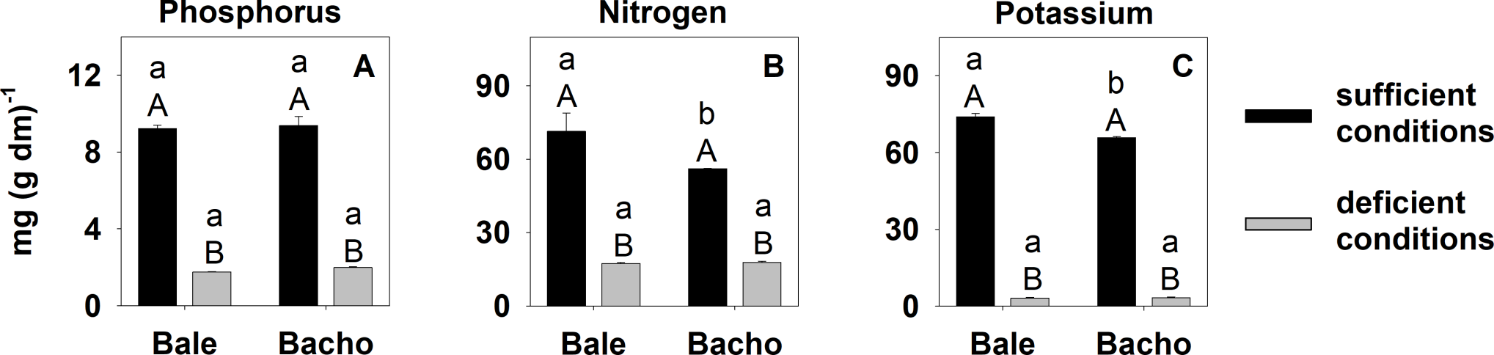


**Supplementary Figure 5.** P (A), N (B) and K (C) concentration in the shoot of *B. carinata* cv. Bale and cv. Bacho affected by nutrient supply. Small letters denote significant differences at P < 0.05 between cv.s at the same nutrient level; capital letters between the treatments of the respective cultivar (Tukey test). Columns represent means and bars SE; n = 3.


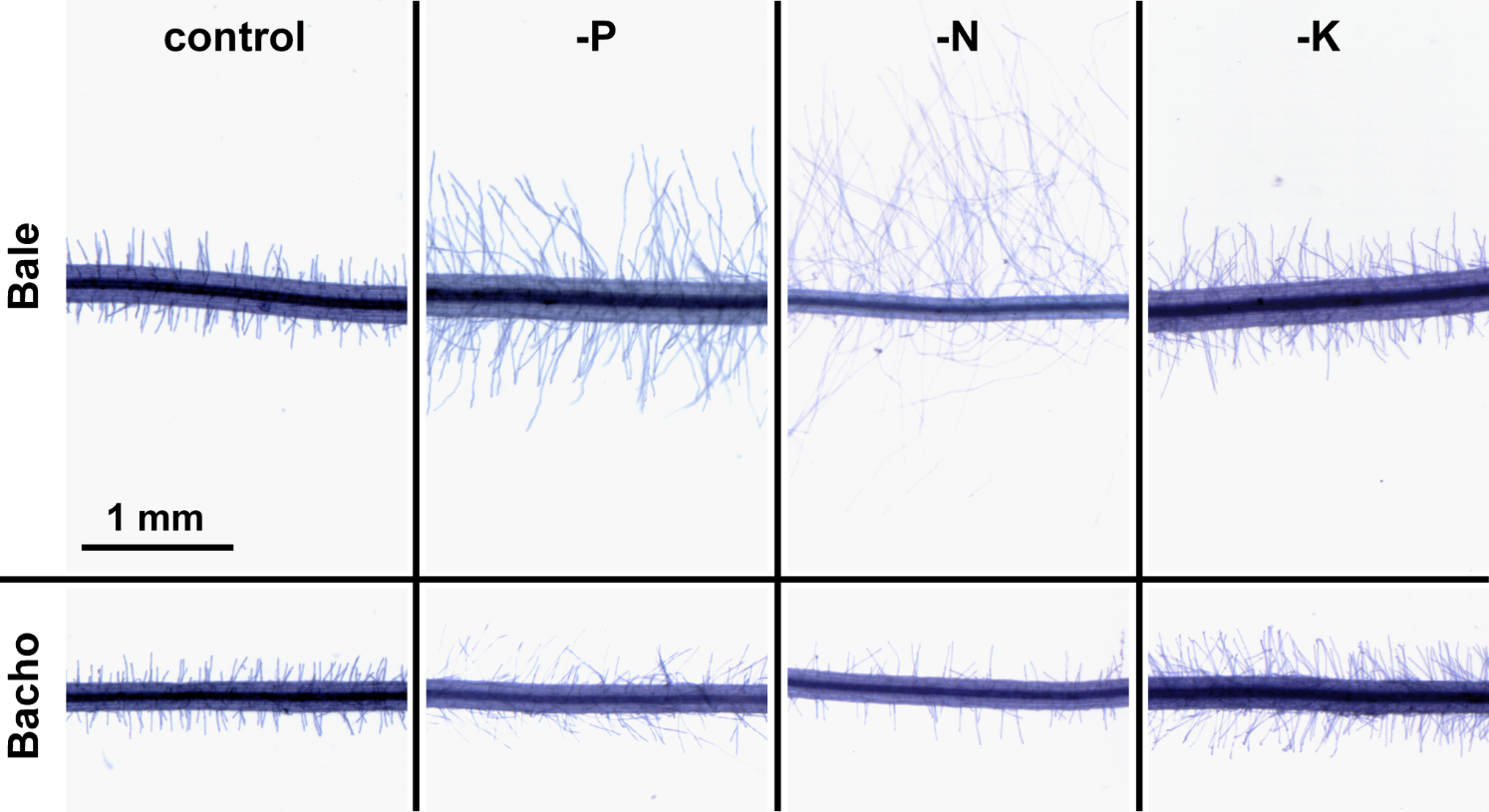


**Supplementary Figure 6.** Representative root hairs of *B. carinata* cv. Bale and cv. Bacho affected by different nutrient deficiencies.


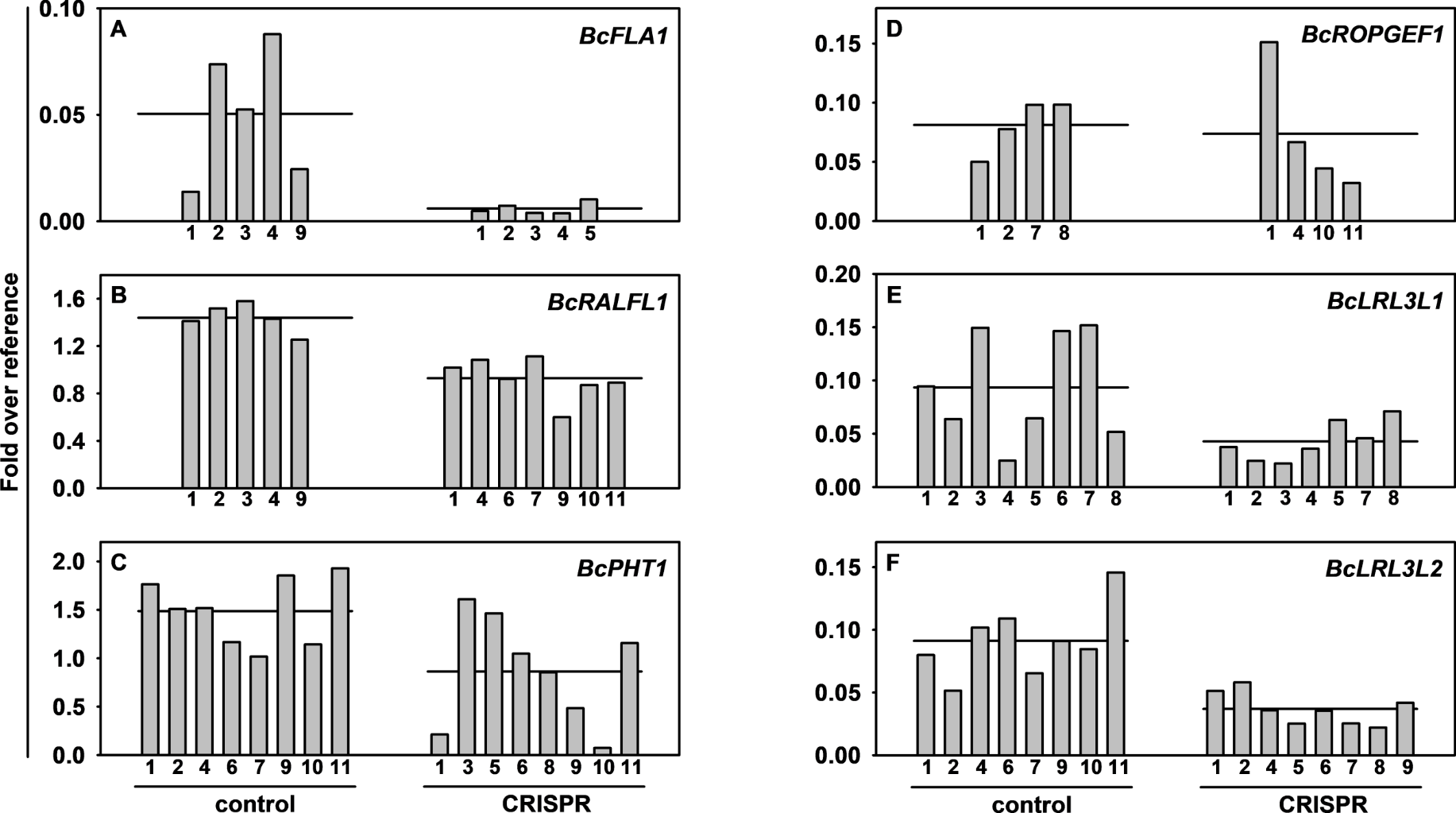


**Supplementary Figure 7.** Expression of the six candidate genes for Pi deficiency-induced root hair elongation (A-F) affected by CRISPR/Cas9-induced gene-editing determined by qPCR. Each column represents the expression in root tips of one single transgenic root measured by three technical replicates with the horizontal line representing the corresponding mean of all biological replicates (all transgenic roots). Fold over reverence = 2^-delta Ct^. Numbers below the columns indicate the respective transgenic roots.


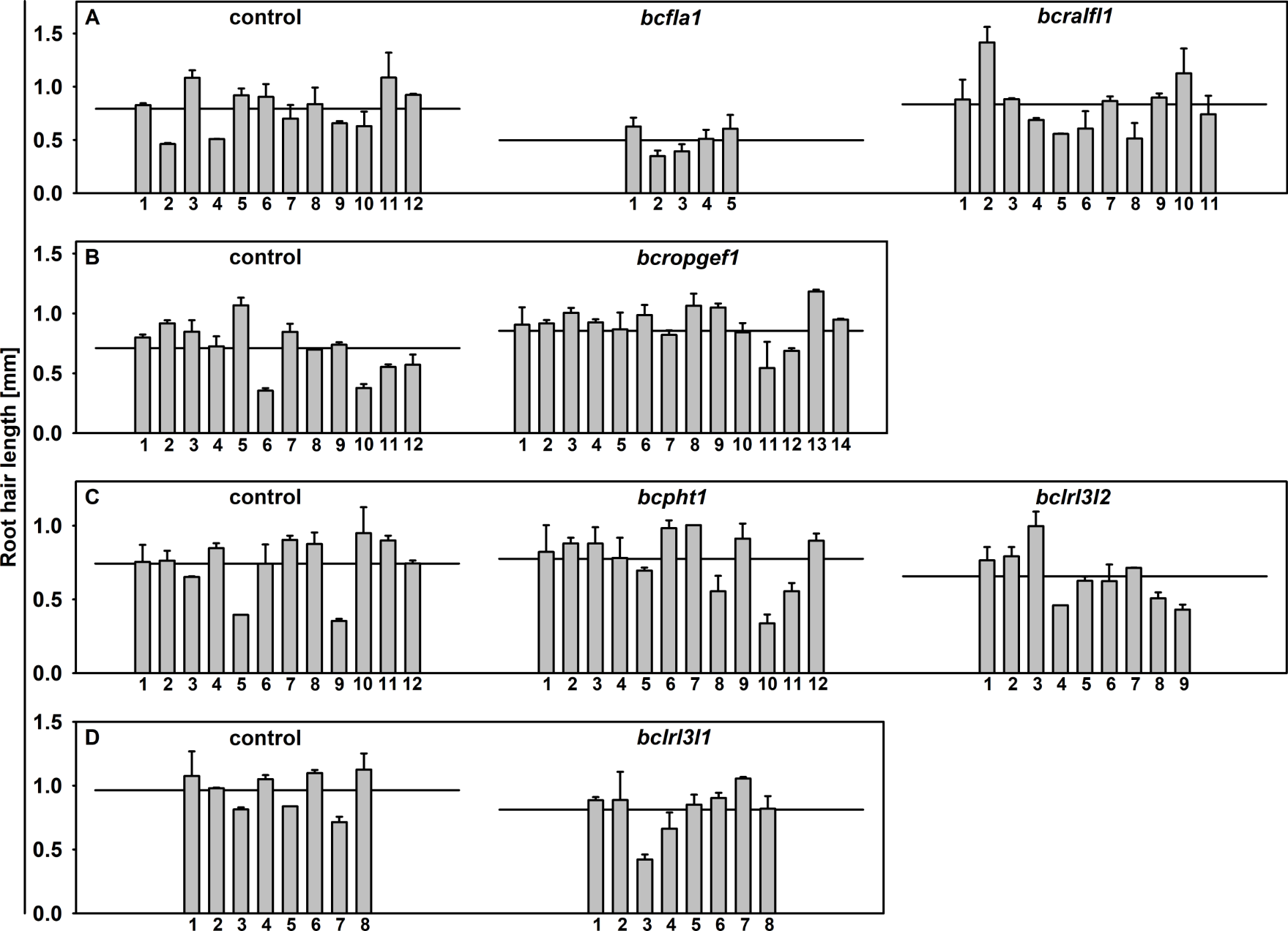


**Supplementary Figure 8.** Root hair length of the transgenic 1^st^ and 2^nd^ order lateral hairy roots after targeting the respective candidate genes for Pi deficiency-induced root hair elongation (A-D) by CRISPR/Cas9. Each column represents the mean of the root hair length of root tips of one single transgenic root with the horizontal line representing the corresponding mean of all biological replicates (all transgenic roots). Bars indicate SE. Numbers below the columns indicate the respective transgenic roots.

# Supplementary Tables

**Supplementary Table 1.** Number of genes in the different annotations of the MACE tags according to the filter steps.

| **annotation**  **filter** | **upregulation** | | **downregulation** | |
| --- | --- | --- | --- | --- |
|  | ***B. rapa***  **(GenXPro)** | ***B. napus***  **(CLC)** | ***B. rapa***  **(GenXPro)** | ***B. napus***  **(CLC)** |
| database entries with dedicated tags | 46,685 | 62,555 | 46,685 | 62,555 |
| at least 2-fold regulated in Bale | 3,796 | 3,550 | 3,419 | 4,062 |
| at least 3 normalized tags in Bale | 1,511 | 1,470 | 884 | 1,170 |
| regulation in Bacho between 0.7 and 1.5-fold | 463 | 411 | 348 | 516 |
| Bale -P at least 1.5-fold higher / lower than Bacho -P | 313 | 270 | 199 | 258 |

**Supplementary Table 2.** Accession numbers of the candidate gene cDNA and gDNA sequences.

| **Gene** | **MACE Seq. Bale** | **MACE Seq. Bacho** | **RACE Seq. Bale** | **gDNA Seq. Bale** |
| --- | --- | --- | --- | --- |
| *BcEF-1-a1* | MF061227 | MG662457 |  |  |
| *BcAGP1* | MG662506 | MG662505 |  |  |
| *BcAGP2* | MG662508 | MG662507 |  |  |
| *BcFLA1a* | MG662461 | MG662460 | KY905141 | KY965932 |
| *BcFLA1b* |  |  | KY905142 | KY965933 |
| *BcCaBP1* | MG662446 | MG662445 |  |  |
| *BcCML25L* | MG662448 | MG662447 |  |  |
| *BcCNGB1L* | MG662450 | MG662449 |  |  |
| *BcRALFL1* | MG662510 | MG662509 | MG682497 | MG686080 |
| *BcCOW1L* | MG662452 | MG662451 |  |  |
| *BcPHT1* | MG662477 | MG662476 | MG682496 |  |
| *BcPHF1* | MG662475 | MG662474 |  |  |
| *BcRLCK1* | MG662487 | MG662486 |  |  |
| *BcMRIL* | MG662473 | MG662472 | MG682495 | MG686077 |
| *BcLRR-RLK1* | MG662469 | MG662468 |  |  |
| *BcIREL* | MG662463 | MG662462 |  |  |
| *BcMRH1L* | MG662471 | MG662470 |  |  |
| *BcRHS10L* | MG662483 | MG662482 |  |  |
| *BcPIP5K1* | MG662479 | MG662478 |  |  |
| *BcROPGEF1* | MG662489 | MG662488 | MG682499 | MG686081 |
| *BcROPGEF2* | MG662491 | MG662490 |  |  |
| *BcAGD1* | MG662442 | MG662441 |  |  |
| *BcEXO70L* | MG662459 | MG662458 |  |  |
| *BcRHSL* | MG662485 | MG662484 | MG682498 |  |
| *BcSHV2L* | MG662493 | MG662492 |  |  |
| *BcSHV3L* | MG662495 | MG662494 |  |  |
| *BcLRL3L1* | MG662465 | MG662464 | MG682493 | MG686078 |
| *BcLRL3L2* | MG662467 | MG662466 | MG682494 | MG686079 |
| *BcWRKY1* | MG662502 | MG662501 |  |  |
| *BcZC3H1* | MG662504 | MG662503 |  |  |
| *BcATL12L* | MG662444 | MG662443 |  |  |
| *BcDUF538* | MG662454 | MG662453 |  |  |
| *BcDUF620* | MG662456 | MG662455 |  |  |
| *BcUNK1* | MG662511 | MG662496 |  |  |
| *BcUNK2* | MG662498 | MG662497 |  |  |
| *BcUNK3* | MG662500 | MG662499 |  |  |
| *BcPP1* | MG662481 | MG662480 |  |  |

**Supplementary Table 3.** qPCR primers. Primers for the candidate genes for Pi deficiency-induced root hair growth and *BcEF-1-a1* were designed on the partial cDNA sequences obtained by MACE. *AtUBC9* primers derived from the study of Czechowski et al. (2005).

| **Gene** | **Sequence forward / reverse (5’ 🡪 3’)** | **Amplicon size** |
| --- | --- | --- |
| *AtUBC9* | TCACAATTTCCAAGGTGCTGC TCATCTGGGTTTGGATCCGT | 61 |
| *BcEF-1-a1* | GGTGACGCTGGTATGGTGAA TGATGACACCGACTGCAACA | 127 |
| *AtIPS2* | TGAGGATTGTGTGTGAATGGA GAACCGAAGCTTGCCAAAGG | 118 |
| *BcAGP1* | CCAGCACCCATCATGGTTCT TAACCGGTGGTGAAGCAACC | 109 |
| *BcAGP2* | TCGCAGTGTTTTGTCTATTGGC TGTTGGTGGTTGGAACGACA | 126 |
| *BcFLA1* | TCGTTGAAACACGTGTCAGT ACTTATCCGATGGAGCCCCT | 189 |
| *BcCaBP1* | GGCGTACCGGCCTATGTTAG TGAAGATAGACGAGGACAAGTGA | 95 |
| *BcCML25L* | GATGAGTGTTCGATCGCGGA ATCACGCCTTGATCCCAACG | 117 |
| *BcCNGB1L* | AGGTAAGAAGGAATCTCCATCAGC CACTTAGATGCGGCAGTTTCA | 138 |
| *BcRALFL1* | AGGAAGAGGAGATGGAGTTCGA TGTTCTTCCTCATCGCACCG | 90 |
| *BcCOW1L* | GCGCTTAACCGTGTTCAAGT CCAAAATCTCCTTCTGGCCCA | 91 |
| *BcPHT1* | CCATCGTGGGAGCTTTTGGT TGACACCAAGCATGATCAGAGA | 117 |
| *BcPHF1* | GTCTTTTTGGAGATTCATCCACTAC ACGTCTCGTGTGGAGTTACATAA | 100 |
| *BcRLCK1* | GCGGCTGACGGATACACTTA ACTCGACAATGGCTGCATGA | 139 |
| *BcMRIL* | TTTCCGGCCTAACATGACCA AGAGAGCCAGAACAACAACAA | 139 |
| *BcLRR-RLK1* | GGAGAATGGAGCTTCACGAGG ACGAAGATCGAAAACTCTCTTGAC | 90 |
| *BcIREL* | TGATCTTGTCCAAAAGAACGCT ACCAATCAGCACTTGAAGTAGA | 147 |
| *BcMRH1L* | CCAGACCAGAGACATCGACC GTTCTGCCCACCAAAGAGGA | 115 |
| *BcRHS10L* | AGTGAAGGGGACATGGGAGA GGACAGAGTAGTCTCCACTGT | 166 |
| *BcPIP5K1* | TGGACCATTAATTAGACTTGGGGA CAACAACCTCCTTCCTTGTATTCTT | 131 |
| *BcROPGEF1* | TGGGAATGACAAGAACCACAT GAGGTCTGAGAGAGTTCAGGG | 90 |
| *BcROPGEF2* | ACTTCATAGGGTGGAGATTGTCA TCACTCGTGCTTCTCATGTTCT | 175 |
| *BcAGD1* | TAAGAGAAACGAGGCGGCTG CCTCTCTCCACTTTTTGAAGCC | 133 |
| *BcEXO70L* | AGAGAGAGACGTGGGGGAAG CCCTCAGCTCACTCTGAAGC | 183 |
| *BcRHSL* | CAACAACAACTGCTCTGGCC TTGGTTCTTGGACCTCTCCG | 105 |
| *BcSHV2L* | AAGCGTATTCGTTCAACGCG GGCACATTAATCCCCGGTGT | 199 |
| *BcSHV3L* | CCGGTGTTCACAGATGCTGA GCTGGTGCATTGGTTGATGG | 113 |
| *BcLRL3L1/2* | CCCATCTCTCTCGCAACTGC TCCATAGGAGAGGAGGCTTCA | 140 |
| *BcWRKY1* | GCCATCACGTCTATTATCAACGG GCTAAAAGGCCATAACTAGAATCCA | 200 |
| *BcZC3H1* | ATCCATTGTGAACCTATGGACA TCTCTACGTAACTGTCGAAATCTC | 96 |
| *BcATL12L* | GAAGCAACAGCGGTTTGGAC GGCAACCACATCCTTCGTCT | 94 |
| *BcDUF538* | AAACTGACGGTGCTGATCCC TACACCTTTGACGAGTCCGC | 185 |
| *BcDUF620* | CATAGCACACGGTGGTCGAA GTTCATATCCGACGGTGGCA | 157 |
| *BcUNK1* | CAATCAAACAACTTCAAGATCCCAC TCAAAAGCTGATCCAATTGCCA | 94 |
| *BcUNK2* | TCTCTGCTCCTGGAACAACC GTCATTGTAATCCATCTTCCTTCCA | 117 |
| *BcUNK3* | AAAACGGTGGTACTTCTCATCA CACCAATTTTGAAGGTGGTACAAGA | 112 |
| *BcPP1* | TGGACGGTTGTTTTGCCTAGA CCAAACGCTCTTGAGACAGC | 96 |

**Supplementary Table 4.** Primers used for 5’ RLM-RACE.

| **Oligo name** | **Primer sequence (5’ 🡪 3’)** |
| --- | --- |
| BcFLA1_RACE_outer | CAATCCATGCATCATATCCAAC |
| BcFLA1_RACE_inner | ACCCAAATCAAACGACGAGT |
| BcCML25L_RACE_outer | TGTGATTCTCCCAAAGAACAAG |
| BcCNGB1L_RACE_outer | ACATGTATCACTTAGATGCGGCAGT |
| BcCNGB1L_RACE_inner | TCTTCCTTGGGTTCTCTTGCA |
| BcRALFL1_RACE_outer | CGCAAGGGCAAAACAGGAAT |
| BcRALFL1_RACE_inner | CTCTTCAACGCCTGCACCT |
| BcCOW1L_RACE_outer | TCCAAAGCCTTCTTGGTCTCA |
| BcCOW1L_RACE_inner | ACTTGAACACGGTTAAGCG |
| BcPHT1_RACE_outer | TGGTCATTTCTCAACCTCAGTCT |
| BcPHT1_RACE_inner | GGACAAGGAAGGTGAAGAGCA |
| BcMIRL_RACE_outer | ACAACAAGAAGGAAAGAGAGG |
| BcMIRL_RACE_inner | AGAGAGCCAGAACAACAACA |
| BcLRR-RLK1_RACE_outer | GCTTACTCATCCACCATAACTAAGA |
| BcLRR-RLK1_RACE_inner | GGTGATCAGTCTTTTACCTACAAAC |
| BcIREL_RACE_outer | TCATACTTCGGGCACAGAGC |
| BcIREL_RACE_inner | GGATTTGGTCAAGGGGCTGA |
| BcRHS10L_RACE_outer | AATGCTGTACAGGGAGCTCA |
| BcRHS10L_RACE_inner | TGAATCAGAACCGCCGGTTAT |
| BcROPGEF1_RACE_outer | CTCGGTTATACTGAATCTTGCAGA |
| BcROPGEF1_RACE_inner | AGGTCTGAGAGAGTTCAGGGA |
| BcROPGEF2_RACE_outer | GTGCCTTCATTATATGTACACACAC |
| BcROPGEF2_RACE_inner | TTAACCTCCCCTACTCTCTCC |
| BcAGD1_RACE_outer | TCTGGATCTCAAGAACACCTCTC |
| BcAGD1_RACE_inner | CCTCTCTCCACTTTTTGAAGCC |
| BcRHSL_RACE_outer | ACATGCCGGTTACTTGTAGA |
| BcRHSL_RACE_inner | GTTCTTCACGCGTTCTGTGC |
| BcSHV2L_RACE_outer | AATTACCAACGCCACAACAA |
| BcSHV2L_RACE_inner | ACCCATTTCTTCTTCCACCACT |
| BcSHV3L_RACE_outer | ACATTATGGTTCCGGCTGTT |
| BcSHV3L_RACE_inner | ACAAAGATGTGTGATCAAGGGT |
| BcLRL3L1_RACE_outer | TGAGAGATCCTATGGTTTAGGC |
| BcLRL3L1_RACE_inner | TATGGTTTAGGCCTTGTTGGA |
| BcLRL3L2_RACE_outer | TTAGCCCTTGGAGGCAGATA |
| BcLRL3L2_RACE_inner | ACATCATCCATAGGAGAGGAGTCT |
| BcWRKY1_RACE_outer | GCTAAAAGGCCATAACTAGAATCCA |
| BcWRKY1_RACE_inner | TGTTATTGCCTGTCACCGCT |
| BcZC3H1_RACE_outer | CTCTAACCTTTATGCTCCTATCTTC |
| BcZC3H1_RACE_inner | CCAGAAAGGGAAGAAAACGGA |
| BcATL12L_RACE_outer | ATCACCATTGGCCAAAACTT |
| BcATL12L_RACE_inner | GTCTAATCTTATTCAGCACGCT |
| BcUNK3_RACE_outer | CACCAATTTTGAAGGTGGTACA |
| BcUNK3_RACE_inner | TCTTGATGAGAAGTACCACCGT |

**Supplementary Table 5.** Primers used for amplification of the Bale gDNA sequences.

| **Gene** | **Sequence forward / reverse (5’ 🡪 3’)** | **Amplicon size** |
| --- | --- | --- |
| *BcFLA1* | TTCAAACGCAATGGCAACCA ACCCAAATCAAACGACGAGTTTC | 752 |
| *BcRALFL* | AGTTTCAGACAGAGAGAGAGAGA CGCAAGGGCAAAACAGGAAT | 500 |
| *BcROPGEF1* | ACTCGATCATTACACAGAGTCTCG CAGAACACAATCATAACCAACACAC | 2470 |
| *BcLRL3L1* | AGAGATGGAGAAGGGGAATGG CCTATGGTTTAGGCCTTGTTGG | 1221 |
| *BcLRL3L2* | AGAGATGGGAAATGAGAATGGAGA GCCCTTGGAGGCAGATACAT | 1371 |

**Supplementary Table 6.** Primers used for construct preparation.

| **Oligo name** | **Primer sequence forward / reverse (5’ 🡪 3’)** |
| --- | --- |
| BcFLA1_gR1 | TAGGTCTCCAGAGGAAGACAGGTTTTAGAGCTAGAA  ATGGTCTCACTCTCCACGGAGTGCACCAGCCGGGAA |
| BcFLA1_gR2 | TAGGTCTCCTCGCCGTGATGTGTTTTAGAGCTAGAA  ATGGTCTCAGCGATCCTTGAATGCACCAGCCGGGAA |
| BcRALFL1_gR1 | TAGGTCTCCTCATCGCGCTCCGTTTTAGAGCTAGAA  ATGGTCTCAATGACGGCGTAGTGCACCAGCCGGGAA |
| BcRALFL1_gR2 | TAGGTCTCCCAACGCAACCGGGTTTTAGAGCTAGAA  ATGGTCTCAGTTGACGGCAGATGCACCAGCCGGGAA |
| BcPHT1_gR1 | TAGGTCTCCGCTTGGTCACCAGTTTTAGAGCTAGAA  ATGGTCTCAAAGCTTCTTGGCTGCACCAGCCGGGAA |
| BcPHT1_gR2 | TAGGTCTCCGGGAAGTGAACCGTTTTAGAGCTAGAA  ATGGTCTCATCCCCCTCATGTTGCACCAGCCGGGAA |
| BcROPGEF1_gR1 | TAGGTCTCCTCTTCACCGTACGTTTTAGAGCTAGAA  ATGGTCTCAAAGAGACGGAGCTGCACCAGCCGGGAA |
| BcROPGEF1_gR2 | TAGGTCTCCGGTGAGATAAACGTTTTAGAGCTAGAA  ATGGTCTCACACCGGTTCGACTGCACCAGCCGGGAA |
| BcLRL3L1_gR1 | TAGGTCTCCGAGATGAGCCCCGTTTTAGAGCTAGAA  ATGGTCTCATCTCTTCCCCCTTGCACCAGCCGGGAA |
| BcLRL3L1_gR2 | TAGGTCTCCGTGAGAGCGTGGGTTTTAGAGCTAGAA  ATGGTCTCATCACCGTCCCCGTGCACCAGCCGGGAA |
| BcLRL3L2_gR1 | TAGGTCTCCAAGGAGGGAGAGGTTTTAGAGCTAGAA  ATGGTCTCACCTTGCTGGGACTGCACCAGCCGGGAA |
| BcLRL3L2_gR2 | TAGGTCTCCAAGAGGCGTCGAGTTTTAGAGCTAGAA  ATGGTCTCATCTTTCCCCGACTGCACCAGCCGGGAA |

**Supplementary Table 7.** Selected candidate genes for the Pi deficiency-induced root hair growth in *Brassica carinata*.

|  |  |  |  | **Tags (normalized to 1 m.)** | | | | **Fold change** | |
| --- | --- | --- | --- | --- | --- | --- | --- | --- | --- |
|  |  |  |  | **Bale** | | **Bacho** | | **Bale -P / +P** | **Bacho -P / +P** |
| **Gene name**  **abbr.** | **Annotated**  **gene id** | ***A. thaliana***  **gene id** | **Description** | **+P** | **-P** | **+P** | **-P** |  |  |
| **Endogenous control** | | | | | | | | | |
| *BcEF-1-a1* | Bra031605 | AT5G60390 | Elongation factor 1 alpha 1 | 58.0 | 55.0 | 55.2 | 55.2 | 0.9 | 1.0 |
| **Arabinogalactan proteins** | | | | | | | | | |
| *BcAGP1* | TC167185 | AT4G40090 | Arabinogalactan protein 1 | 144.5 | 857.6 | 190.9 | 180.8 | 5.9 | 0.9 |
| *BcAGP2* | Bra025551 | AT5G40730 | Arabinogalactan protein 2 | 80.4 | 316.7 | 105.9 | 110.8 | 3.9 | 1.0 |
| *BcFLA1* | Bra033722 | AT5G44130 | Fasciclin-like arabinogalactan protein 1 | 53.4 | 116.1 | 26.9 | 22.3 | 2.2 | 0.8 |
| **Calcium-related proteins** | | | | | | | | | |
| *BcCaBP1* | Bra032925 | AT1G29020 | Calcium-binding EF-hand family protein 1 | 1.2 | 3.8 | 0.8 | 0.9 | 3.1 | 1.2 |
| *BcCML25L* | TC189239 | AT1G24620 | Calmodulin-like 25-like | 2.3 | 8.0 | 1.8 | 2.4 | 3.5 | 1.4 |
| *BcCNGB1L* | Bra009801  ES266808 | AT5G24880 | Cyclic nucleotide-gated cation channel beta-1-like | 11.1  4.1 | 41.1  20.0 | 8.8  4.7 | 12.9  6.6 | 3.7  4.9 | 1.5  1.4 |
| *BcRALFL1* | Bra001149  TC167929 | AT3G05490 | Rapid alkalinization factor-like 1 | 116.9  109.4 | 269.8  230.7 | 154.4  56.0 | 160.4  60.6 | 2.3  2.1 | 1.0  1.1 |
| *BcCOW1L* | ES907398 | AT4G34580 | Can of worms 1-like | 4.7 | 34.7 | 10.0 | 12.3 | 7.4 | 1.2 |
| **Phosphate transport** | | | | | | | | | |
| *BcPHT1* | Bra027492 | AT5G43360 | Inorganic phosphate transporter 1 | 3.2 | 49.8 | 5.1 | 5.5 | 15.8 | 1.1 |
| *BcPHF1* | TC192478 | AT3G52190 | Phosphate transporter traffic facilitator 1 | 35.5 | 71.0 | 20.8 | 30.6 | 2.0 | 1.5 |
| **Protein kinases** | | | | | | | | | |
| *BcRLCK1* | Bra022543 | AT5G51270 | Receptor-like cytoplasmic kinase 1 | 3.3 | 9.7 | 3.2 | 3.3 | 2.9 | 1.0 |
| *BcMRIL* | Bra004665 | AT2G41970 | Receptor-like cytoplasmic kinase MARIS-like | 4.9 | 13.0 | 4.4 | 4.1 | 2.7 | 0.9 |
| *BcLRR-RLK1* | Bra011255 | AT4G31250 | Leucine-rich repeat receptor-like serine/threonine protein kinase 1 | 3.9 | 16.0 | 3.7 | 3.9 | 4.1 | 1.1 |
| *BcIREL* | Bra010074 | AT5G62310 | Incomplete root hair elongation-like (serine/threonine protein kinase) | 1.3 | 9.4 | 2.0 | 2.7 | 7.3 | 1.3 |
| *BcMRH1L* | TC212815 | AT4G18640 | Morphogenesis of root hair 1-like (serine/threonine protein kinase) | 22.6 | 46.1 | 23.9 | 28.6 | 2.0 | 1.2 |
| *BcRHS10L* | Bra007915 | AT1G70460 | Root hair specific 10-like (proline-rich extensin-like receptor kinase) | 6.3 | 15.9 | 6.8 | 5.3 | 2.5 | 0.8 |
| *BcPIP5K1* | Bra007756 | AT2G26420 | Phosphatidylinositol phosphate 5-kinase 1 | 4.8 | 24.0 | 2.6 | 6.0 | 4.9 | 2.3 |
| **GTPase-related** | | | | | | | | | |
| *BcROPGEF1* | Bra004945 | AT2G45890 | Rop guanine nucleotide exchange factor 1 | 10.1 | 24.8 | 9.4 | 11.1 | 2.4 | 1.2 |
| *BcROPGEF2* | Bra020048 | AT5G19560 | Rop guanine nucleotide exchange factor 2 | 1.7 | 16.3 | 1.6 | 1.8 | 9.3 | 1.1 |
| *BcAGD1* | Bra001277 | AT3G07940 | Adenosine diphosphate (ADP) ribosylation factor (ARF)-GTPase activating protein (GAP) domain-containing protein 1 | 3.0 | 10.5 | 3.8 | 4.1 | 3.4 | 1.1 |
| *BcEXO70L* | TC204552 | AT5G13990 | Exocyst complex component EXO70-like | 6.7 | 23.1 | 11.3 | 11.7 | 3.4 | 1.0 |
| **Root hair affecting** | | | | | | | | | |
| *BcRHSL* | Bra005382 | AT2G34910 | Root hair specific-like | 4.5 | 24.0 | 9.6 | 10.4 | 5.4 | 1.1 |
| *BcSHV2L* | Bra036147 | AT5G49270 | Shaven 2-like | 13.6 | 48.4 | 12.9 | 16.2 | 3.6 | 1.3 |
| *BcSHV3L* | Bra019092 | AT4G26690 | Shaven 3-like (glycerophosphodiester phosphodiesterase-like, GDPDL) | 8.9 | 31.0 | 15.3 | 17.8 | 3.5 | 1.2 |
| **Transcription factors** | | | | | | | | | |
| *BcLRL3L1* | Bra006788 | AT5G58010 | *Lotus japonicus* roothairless1-like 3 (LRL3)-like 1 | 8.5 | 24.2 | 7.5 | 8.5 | 2.9 | 1.1 |
| *BcLRL3L2* | Bra020398 | AT5G58010 | *Lotus japonicus* roothairless1-like 3 (LRL3)-like 2 | 7.2 | 21.0 | 8.8 | 10.8 | 2.9 | 1.2 |
| *BcWRKY1* | Bra029491 | AT4G04450 | WRKY transcription factor 1 | 1.0 | 4.7 | 1.6 | 1.1 | 4.5 | 0.7 |
| **Zinc finger proteins** | | | | | | | | | |
| *BcZC3H1* | Bra013181 | AT2G05160 | Zinc finger CCCH domain-containing protein 1 | 2.4 | 6.7 | 2.1 | 2.6 | 2.8 | 1.3 |
| *BcATL12L* | TC178182 | AT2G20030 | Arabidopsis Tóxicos en Levadura (ATL) 12-like  (RING-H2 finger protein) | 6.2 | 19.9 | 7.6 | 7.7 | 3.2 | 1.0 |
| **Unknown** | | | | | | | | | |
| *BcDUF538* | TC174694 | AT5G01610 | Protein containing domain of unknown function (DUF) 538 | 2.6 | 10.8 | 2.0 | 2.6 | 4.1 | 1.3 |
| *BcDUF620* | Bra008179 | AT1G75160 | Protein containing domain of unknown function (DUF) 620 | 0.2 | 5.5 | 0.4 | 0.4 | 28.3 | 1.2 |
| *BcUNK1* | Bra028503 ES907952 | AT5G41761 | Unknown protein 1 | 0.4  0.8 | 13.1  28.8 | 0.1  0.1 | 0.2  0.2 | 33.9  34.2 | 1.1  1.3 |
| *BcUNK2* | EE450922 | AT5G43230 | Unknown protein 2 | 3.4 | 22.6 | 3.1 | 4.3 | 6.7 | 1.4 |
| *BcUNK3* | Bra009749 | AT5G24313 | Unknown protein 3 | 2.6 | 19.6 | 0.0 | 0.0 | 7.6 | 0.9 |
| **Protein phosphatases** | | | | | | | | | |
| *BcPP1* | Bra022179 | AT3G16800 | Protein phosphatase 1 | 33.6 | 15.8 | 32.5 | 32.6 | 0.5 | 1.0 |

**Supplementary Table 8.** Relative expression of candidate genes for increased root hair length under Pi deficiency in *B. carinata* cv. Bale and cv. Bacho affected by P supply determined by qPCR in samples obtained from the MACE (1^st^) experiment. *AtUBC9* was used as an endogenous control. Significance was calculated according to Steibel et al. (2009). Stars denote significant differences compared to +P within the cultivar (significance codes: 0 ‘***’ 0.001 ‘**’ 0.01 ‘*’ 0.05).

| **Gene name**  **(abbr.)** | **Relative expression** | | | |
| --- | --- | --- | --- | --- |
|  | **Bale** | | **Bacho** | |
|  | **+P** | **-P** | **+P** | **-P** |
| **Arabinogalactan proteins** | | | | |
| *BcAGP1* | 1.00 | ***4.83 | 1.57 | 0.97 |
| *BcAGP2* | 1.00 | ***3.06 | 1.14 | 1.34 |
| *BcFLA1* | 1.00 | 1.22 | 0.91 | 0.66 |
| **Calcium-related** | | | | |
| *BcCaBP1* | 1.00 | 0.75 | 1.25 | 0.94 |
| *BcCML25L* | 1.00 | 1.06 | 0.71 | ***0.47 |
| *BcCNGB1L* | 1.00 | ***1.85 | 0.73 | 0.75 |
| *BcRALFL1* | 1.00 | ***1.73 | 1.24 | 0.99 |
| *BcCOW1L* | 1.00 | ***2.39 | 1.85 | 1.58 |
| **Phosphate transport** | | | | |
| *BcPHT1* | 1.00 | ***7.55 | 1.33 | ***0.30 |
| *BcPHF1* | 1.00 | ***2.18 | 1.54 | 2.17 |
| **Protein kinases** | | | | |
| *BcRLCK1* | 1.00 | **1.35 | 0.73 | *0.57 |
| *BcMRIL* | 1.00 | 1.25 | 1.90 | *1.41 |
| *BcLRR-RLK1* | 1.00 | 1.32 | 0.75 | 0.62 |
| *BcIREL* | 1.00 | 1.45 | 0.70 | 0.65 |
| *BcMRH1L* | 1.00 | ***1.43 | 1.09 | 1.03 |
| *BcRHS10L* | 1.00 | *1.39 | 1.55 | ***0.94 |
| *BcPIP5K1* | 1.00 | ***2.34 | 0.53 | ***0.73 |
| **GTPase-related** | | | | |
| *BcROPGEF1* | 1.00 | ***1.80 | 1.00 | 0.94 |
| *BcROPGEF2* | 1.00 | ***4.83 | 1.57 | 0.97 |
| *BcAGD1* | 1.00 | 1.13 | 0.84 | 0.40 |
| *BcEXO70L* | 1.00 | 0.84 | 0.81 | 0.57 |
| **Root hair affecting** | | | | |
| *BcRHSL* | 1.00 | ***2.53 | 2.18 | 1.62 |
| *BcSHV2L* | 1.00 | ***2.24 | 1.45 | 1.32 |
| *BcSHV3L* | 1.00 | ***1.84 | 0.81 | ***0.67 |
| **Transcription factors** | | | | |
| *BcLRL3L1/2* | 1.00 | ***2.06 | 1.73 | 1.56 |
| *BcWRKY1* | 1.00 | 1.00 | 0.56 | **0.31 |
| **Zinc finger proteins** | | | | |
| *BcZC3H1* | 1.00 | ***1.73 | 1.06 | 0.91 |
| *BcATL12L* | 1.00 | 1.45 | 1.95 | 1.42 |
| **Unknown** | | | | |
| *BcDUF538* | 1.00 | ***3.21 | 2.59 | 3.24 |
| *BcDUF620* | 1.00 | 1.22 | 0.41 | 0.31 |
| *BcUNK1* | 1.00 | ***7.00 | 0.04 | ***0.02 |
| *BcUNK2* | 1.00 | ***1.81 | 1.22 | 1.18 |
| *BcUNK3* | 1.00 | ***4.28 | 0.00 | 0.00 |
| **Protein phosphatases** | | | | |
| *BcPP1* | 1.00 | ***0.39 | 0.44 | ***0.28 |

**Supplementary Table 9.** Relative expression of candidate genes for increased root hair length under Pi deficiency in *B. carinata* cv. Bale and cv. Bacho affected by P supply determined by qPCR in samples obtained from the independently conducted 2^nd^ experiment. *AtUBC9* was used as an endogenous control. Significance was calculated according to Steibel et al. (2009). Stars denote significant differences compared to +P within the cultivar (significance codes: 0 ‘***’ 0.001 ‘**’ 0.01 ‘*’ 0.05).

| **Gene name**  **(abbr.)** | **Relative expression** | | | |
| --- | --- | --- | --- | --- |
|  | **Bale** | | **Bacho** | |
|  | **+P** | **-P** | **+P** | **-P** |
| **Arabinogalactan proteins** | | | | |
| *BcAGP1* | 1.00 | ***4.05 | 1.13 | 0.82 |
| *BcAGP2* | 1.00 | ***6.06 | 1.29 | ***2.79 |
| *BcFLA1* | 1.00 | ***6.25 | 4.34 | 4.41 |
| **Calcium-related** | | | | |
| *BcCaBP1* | 1.00 | ***4.14 | 6.20 | 7.68 |
| *BcCML25L* | 1.00 | ***2.26 | 1.22 | 1.38 |
| *BcCNGB1L* | 1.00 | *1.98 | 1.02 | 1.06 |
| *BcRALFL1* | 1.00 | ***2.36 | 1.80 | 1.64 |
| *BcCOW1L* | 1.00 | ***2.22 | 1.57 | 1.57 |
| **Phosphate transport** | | | | |
| *BcPHT1* | 1.00 | ***12.00 | 1.67 | ***0.58 |
| *BcPHF1* | 1.00 | ***2.33 | 1.99 | *3.30 |
| **Protein kinases** | | | | |
| *BcRLCK1* | 1.00 | 1.55 | 0.81 | 0.85 |
| *BcMRIL* | 1.00 | ***2.59 | 3.46 | 4.46 |
| *BcLRR-RLK1* | 1.00 | ***1.81 | 0.88 | 0.87 |
| *BcIREL* | 1.00 | ***2.98 | 1.66 | 1.68 |
| *BcMRH1L* | 1.00 | 1.26 | 1.36 | 1.17 |
| *BcRHS10L* | 1.00 | 1.73 | 1.61 | 1.30 |
| **GTPase-related** | | | | |
| *BcROPGEF1* | 1.00 | ***2.65 | 1.47 | 1.55 |
| *BcROPGEF2* | 1.00 | ***5.31 | 1.38 | 1.74 |
| *BcAGD1* | 1.00 | **2.30 | 1.21 | 0.97 |
| *BcEXO70L* | 1.00 | *2.26 | 4.49 | 3.09 |
| **Root hair affecting** | | | | |
| *BcRHSL* | 1.00 | ***6.29 | 4.83 | 5.57 |
| *BcSHV2L* | 1.00 | ***4.02 | 2.05 | 3.02 |
| *BcSHV3L* | 1.00 | 1.69 | 0.93 | 0.82 |
| **Transcription factors** | | | | |
| *BcLRL3L1/2* | 1.00 | ***2.75 | 1.26 | 1.81 |
| *BcWRKY1* | 1.00 | ***6.08 | 1.70 | 1.92 |
| **Zinc finger proteins** | | | | |
| *BcZC3H1* | 1.00 | **2.05 | 1.06 | 1.31 |
| *BcATL12L* | 1.00 | ***3.04 | 2.45 | 3.23 |
| **Unknown** | | | | |
| *BcDUF538* | 1.00 | ***5.62 | 2.82 | ***6.16 |
| *BcDUF620* | 1.00 | ***6.55 | 1.08 | ***2.03 |
| *BcUNK1* | 1.00 | ***37.13 | 0.01 | ***0.02 |
| *BcUNK2* | 1.00 | ***2.64 | 2.02 | 2.40 |
| *BcUNK3* | 1.00 | ***10.19 | 0.00 | 0.00 |
| **Protein phosphatases** | | | | |
| *BcPP1* | 1.00 | ***0.19 | 0.41 | ***0.19 |

**Supplementary Table 10.** Relative expression of candidate genes for increased root hair length under Pi deficiency in *B. carinata* cv. Bale affected by P supply in a split-root system determined by qPCR. *AtUBC9* was used as an endogenous control. Significance was calculated according to Steibel et al. (2009). Different letters denote significant differences between the treatments.

| **Gene name**  **(abbr.)** | **Relative expression** | | | |
| --- | --- | --- | --- | --- |
|  | **+ +** | **+** | **-** | **- -** |
| **Arabinogalactan proteins** | | | | |
| *BcAGP1* | ^b^1.00 | ^b^1.43 | ^b^2.12 | ^a^12.05 |
| *BcAGP2* | ^b^1.00 | ^b^0.94 | ^b^1.10 | ^a^5.10 |
| *BcFLA1* | ^b^1.00 | ^b^0.97 | ^ab^2.12 | ^a^5.31 |
| **Calcium-related** | | | | |
| *BcCaBP1* | ^b^1.00 | ^b^1.12 | ^a^5.62 | ^a^6.63 |
| *BcCML25L* | ^b^1.00 | ^b^1.52 | ^a^5.07 | ^a^7.64 |
| *BcCNGB1L* | ^c^1.00 | ^c^1.24 | ^b^2.29 | ^a^4.12 |
| *BcRALFL1* | ^b^1.00 | ^b^1.00 | ^b^1.12 | ^a^2.42 |
| *BcCOW1L* | ^c^1.00 | ^bc^1.30 | ^b^1.93 | ^a^5.30 |
| **Phosphate transport** | | | | |
| *BcPHT1* | ^c^1.00 | ^b^2.24 | ^b^4.02 | ^a^30.91 |
| **Protein kinases** | | | | |
| *BcRLCK1* | ^b^1.00 | ^b^1.24 | ^a^3.12 | ^a^5.08 |
| *BcMRIL* | ^b^1.00 | ^b^1.40 | ^a^3.96 | ^a^6.29 |
| *BcLRR-RLK1* | ^b^1.00 | ^b^1.18 | ^a^3.24 | ^a^5.78 |
| *BcIREL* | ^b^1.00 | ^b^1.25 | ^a^4.39 | ^a^7.66 |
| *BcMRH1L* | ^c^1.00 | ^bc^1.16 | ^b^1.64 | ^a^3.02 |
| *BcRHS10L* | ^c^1.00 | ^c^1.10 | ^b^1.97 | ^a^3.58 |
| *BcPIP5K1* | ^b^1.00 | ^b^1.19 | ^a^3.13 | ^a^6.20 |
| **GTPase-related** | | | | |
| *BcROPGEF1* | ^c^1.00 | ^c^1.32 | ^b^2.91 | ^a^5.70 |
| *BcROPGEF2* | ^c^1.00 | ^c^1.48 | ^b^3.82 | ^a^9.62 |
| *BcAGD1* | ^b^1.00 | ^b^1.34 | ^a^5.07 | ^a^7.47 |
| *BcEXO70L* | ^b^1.00 | ^b^1.18 | ^a^6.82 | ^a^7.48 |
| **Root hair affecting** | | | | |
| *BcRHSL* | ^c^1.00 | ^c^1.30 | ^b^2.85 | ^a^7.23 |
| *BcSHV2L* | ^c^1.00 | ^bc^1.43 | ^b^2.30 | ^a^6.42 |
| *BcSHV3L* | ^b^1.00 | ^b^1.07 | ^b^1.16 | ^a^2.83 |
| **Transcription factors** | | | | |
| *BcLRL3L1/2* | ^c^1.00 | ^bc^1.21 | ^b^2.22 | ^a^5.05 |
| *BcWRKY1* | ^b^1.00 | ^b^1.14 | ^a^3.00 | ^a^4.40 |
| **Zinc finger proteins** | | | | |
| *BcZC3H1* | ^b^1.00 | ^b^0.98 | ^a^2.43 | ^a^4.18 |
| *BcATL12L* | ^b^1.00 | ^b^1.32 | ^a^4.49 | ^a^6.87 |
| **Unknown** | | | | |
| *BcDUF538* | ^c^1.00 | ^bc^1.10 | ^b^2.60 | ^a^6.61 |
| *BcUNK1* | ^c^1.00 | ^c^1.46 | ^b^5.54 | ^a^25.83 |
| *BcUNK2* | ^b^1.00 | ^b^1.51 | ^a^4.15 | ^a^8.67 |
| *BcUNK3* | ^c^1.00 | ^bc^2.01 | ^b^4.74 | ^a^24.79 |

**Supplementary Table 11.** Relative expression of candidate genes for increased root hair length under Pi deficiency in *B. carinata* cv. Bale and cv. Bacho affected by nutrient deficiency determined by qPCR. *AtUBC9* and *BcEF-1-a1* were used as endogenous controls. Significance was calculated according to Steibel et al. (2009). Stars denote significant differences compared to the control (C) within the cultivar (significance codes: 0 ‘***’ 0.001 ‘**’ 0.01 ‘*’ 0.05).

| **Gene name**  **(abbr.)** | **Relative expression** | | | | | | | |
| --- | --- | --- | --- | --- | --- | --- | --- | --- |
|  | **Bale** | | | | **Bacho** | | | |
|  | **C** | **-P** | **-N** | **-K** | **C** | **-P** | **-N** | **-K** |
| **Arabinogalactan proteins** | | | | | | | | |
| *BcAGP1* | 1.00 | ***6.52 | ***3.02 | 0.97 | 1.52 | 1.33 | 1.64 | 1.57 |
| *BcAGP2* | 1.00 | ***5.24 | 1.93 | 0.86 | 2.35 | 3.61 | 1.66 | 1.19 |
| *BcFLA1* | 1.00 | ***3.98 | 1.09 | 0.79 | 3.72 | 2.69 | 2.95 | 3.03 |
| **Calcium-related** | | | | | | | | |
| *BcCaBP1* | 1.00 | ***5.08 | *3.28 | 1.21 | 5.70 | 8.77 | 7.86 | 7.35 |
| *BcCML25L* | 1.00 | 1.96 | 1.81 | 0.75 | 0.97 | 0.94 | 1.04 | 1.04 |
| *BcCNGB1L* | 1.00 | ***3.63 | ***2.15 | 1.40 | 1.49 | 1.93 | *2.57 | 1.87 |
| *BcRALFL1* | 1.00 | **1.99 | 1.08 | 0.82 | 1.41 | 1.88 | 1.41 | 1.13 |
| *BcCOW1L* | 1.00 | ***3.94 | *2.16 | 1.07 | 2.44 | 2.85 | 2.51 | 2.84 |
| **Phosphate transport** | | | | | | | | |
| *BcPHT1* | 1.00 | ***20.55 | 2.08 | 1.35 | 3.54 | 2.70 | *1.57 | 3.41 |
| **Protein kinases** | | | | | | | | |
| *BcRLCK1* | 1.00 | ***3.69 | *2.37 | 0.93 | 1.38 | 1.44 | 1.61 | 1.48 |
| *BcMRIL* | 1.00 | 1.61 | 0.75 | 0.59 | 2.25 | 2.92 | 2.22 | 2.01 |
| *BcLRR-RLK1* | 1.00 | ***3.12 | 1.48 | 0.72 | 1.06 | 1.55 | 1.20 | 1.28 |
| *BcIREL* | 1.00 | ***4.88 | **2.41 | 1.20 | 2.27 | 2.67 | 2.82 | 2.85 |
| *BcMRH1L* | 1.00 | **2.11 | 1.58 | 1.76 | 1.97 | 2.38 | 3.01 | 3.16 |
| *BcRHS10L* | 1.00 | ***2.88 | 1.87 | 1.17 | 1.89 | 1.47 | 2.71 | 2.82 |
| *BcPIP5K1* | 1.00 | ***5.53 | ***3.41 | 1.70 | 1.23 | 2.14 | 1.98 | 1.63 |
| **GTPase-related** | | | | | | | | |
| *BcROPGEF1* | 1.00 | ***2.17 | 1.08 | 0.76 | 1.14 | 1.58 | 1.27 | 1.22 |
| *BcROPGEF2* | 1.00 | ***7.83 | *2.98 | 1.53 | 2.43 | 2.44 | 1.88 | 4.29 |
| *BcAGD1* | 1.00 | ***3.61 | 1.81 | 1.43 | 1.84 | 2.39 | 2.47 | 2.52 |
| *BcEXO70L* | 1.00 | *3.53 | *4.22 | 2.15 | 3.29 | 3.34 | 6.22 | 4.88 |
| **Root hair affecting** | | | | | | | | |
| *BcRHSL* | 1.00 | *3.30 | 1.47 | 0.88 | 2.89 | 3.54 | 2.70 | 4.58 |
| *BcSHV2L* | 1.00 | ***5.62 | **3.39 | 1.59 | 3.49 | 4.51 | 3.22 | 2.88 |
| *BcSHV3L* | 1.00 | ***5.04 | *2.24 | 2.06 | 2.09 | 2.55 | 1.72 | 2.84 |
| **Transcription factors** | | | | | | | | |
| *BcLRL3L1/2* | 1.00 | ***2.39 | 0.93 | 0.84 | 1.44 | 1.75 | 1.00 | 1.34 |
| *BcWRKY1* | 1.00 | 3.96 | **7.55 | 4.38 | 1.68 | 2.20 | 3.81 | 3.15 |
| **Zinc finger proteins** | | | | | | | | |
| *BcZC3H1* | 1.00 | 1.16 | 0.94 | 0.57 | 0.60 | 0.70 | 0.82 | 0.97 |
| *BcATL12L* | 1.00 | 1.62 | 1.39 | 1.19 | 2.39 | 3.01 | 2.59 | 2.80 |
| **Unknown** | | | | | | | | |
| *BcDUF538* | 1.00 | ***3.29 | 1.81 | 0.89 | 2.09 | 3.43 | 1.98 | 2.29 |
| *BcUNK1* | 1.00 | ***34.56 | 8.97 | 1.26 | 0.04 | 0.06 | 0.07 | 0.12 |
| *BcUNK2* | 1.00 | ***3.74 | 1.82 | 0.63 | 2.17 | 3.19 | 2.64 | 2.27 |
| *BcUNK3* | 1.00 | **13.86 | 2.64 | 1.15 | 0.00 | 0.00 | 0.00 | 0.00 |

**Supplementary Table 12.** Amino acid sequences encoded by the two *BcFLA1* alleles. Colors indicate motifs identified. N-terminal signal peptide (green) was identified with SignalP 4.1 (Petersen et al., 2011), putative AG glycomodules (red) were marked according to Ma et al. (2017), fasciclin domain (yellow) was marked as in Jun and Xiaoming (2012) and GPI-anchor (purple) was identified with PredGPI (Pierleoni et al., 2008).

| **BcFLA1a** | MATIHLTLAPLLILAAVFLSTEITAQPAAPAPGPAAPTNITAILEKGGQFATFIHLLNTTRVGNQINIQVNSSSEGMTVFAPTDNAFQNLKPGTLNKLSYDDQVKLILYHVSPKFYTLDDLLSVSNPVRTQASGRDSAVYGLNFTSQGNQVNVSTGFVETRVSNALRKERPLAVYVVDMVLLPEEMFGENKVSPAAPGPKSNSPDVSDDQEKAGAPSDKSGSGEMKAGLGLALGLVFLCLKLVV |
| --- | --- |
| **BcFLA1b** | MATSHLTLAPLLILATVLLSTEVTAQPAAPAPGPAGPVNITAILEKGGQFATLIRLLNTTQVGNQINIQVNSSSEGMTVFAPTDNAFQNLKPGTLNKLSHEDQVKLILYHVSPKFYTLDDLLSVSNPVRTQASGRDGAVYGLNFTSQGNQVNVSTGVVETRVSNELRKERPLAIYVVDMVLLPEEMFGENKVSPAAPGPKSNSPDVSDDQEKAGAPSDKSGSSEMKAGLGFALGVVFLCLKLVV |

# Supplementary Text

**Supplementary Text 1.** Description of the candidate genes selected for knockout.

**Arabinogalactan proteins** (AGPs) are extracellular glycoproteins consisting of a core protein, one or more AG side chains and most often a glycosylphosphatidylinositol (GPI) anchor. Among others, they may be involved in cell wall synthesis and remodeling. Their regulatory function could be based on their ability to act as modulators or coreceptors and to serve as a source of small signaling molecules, for example, by the cleavage of the GPI anchor, thus, producing lipid signals. Because of their amphiphilicity, they could mediate between the cytoplasm, the plasma membrane and the cell wall (Seifert and Roberts, 2007). Marzec et al. (2015) have already shown the involvement of AGPs in root hair development in barley. However, the authors concluded that they are involved more in an earlier stage of root hair development than in the elongation stage. One of the three AGPs, *BcFLA1*, was annotated to *BrFLA27* (*AtFLA13*) and belongs to a group of chimeric AGPs, since it has a fasciclin domain between two regions with AGP motifs (Supplementary Table 13). Furthermore, it possesses a N-terminal signal peptide with a cleavage site between position 25 and 26 as well as a C-terminal GPI anchor. The FLAs are thought to play important roles during plant development and in abiotic stress responses and may be involved in interactions within the extracellular matrix (Johnson et al., 2003).

**Calcium** also has an important role in signaling and a central role in root hair elongation forming a calcium gradient (Bibikova et al., 1997; Wymer et al., 1997; Bibikova et al., 1999). *BcRALFL1* belongs to a family of small signaling peptides named the Rapid Alkalinization Factor (RALF) (Murphy and De Smet, 2014). The family diverged into four major groups (Campbell and Turner, 2017) with *BcRALFL1* belonging to group I. The RALF peptides can affect the pH of the extracellular matrix through different pathways and, therefore, have influence on the cell elongation. In addition to their ability to induce MITOGEN-ACTIVATED PROTEIN (MAP) KINASE activity, they can bind to the FER receptor, which leads to a subsequent inhibition of a H^+^-ATPase located in the plasma membrane, so that the apoplastic pH is increased and the cell elongation is inhibited (Haruta et al., 2014; Murphy and De Smet, 2014). Because *AtRALFL22*, to which *BcRALFL1* was annotated, is known to be able to increase the cytosolic Ca^2+^ concentration, the effect on the pH could also be a secondary response via Ca^2+^ signaling (Morato do Canto et al., 2014; Murphy and De Smet, 2014). Furthermore, members of the RALF family seem to be able to regulate the root hair pH and, therefore, elongation. Silencing of *NaRALF* in *Nicotiana attenuata* increased the apoplastic pH at the root hair tip, which led to shorter or even disrupted root hairs, most probably because the cell expansion could not be controlled as is necessary during the transition from the initiation to the elongation (Wu et al., 2007).

Furthermore, two genes involved in **Pi transport** were selected, since Pi itself could serve as a signaling molecule (Chiou and Lin, 2011). The regulation in the MACE could be validated only for *BcPHT1* encoding an inorganic phosphate transporter, whose relative in Arabidopsis (*AtPHT1;3*) is expressed in the epidermis and the pericycle of the main root as well as in trichoblasts of lateral roots (Mudge et al., 2002; Młodzińska and Zboińska, 2016). AtPHT1;3 is known to be induced by low Pi concentrations (Mudge et al., 2002) and contributes to Pi uptake under these conditions, whereas its contribution to Pi uptake under sufficient Pi conditions is negligible (Ayadi et al., 2015). Unexpectedly, in this work, *BcPHT1* was upregulated under Pi deficiency only in Bale, while it was downregulated in Bacho. This indicates that *BcPHT1* may have another or an additional role apart from the Pi uptake in Bale. This is supported by the existence of isoforms of *BcPHT1* in the MACE dataset, which exhibited an upregulation in both cv.s and, therefore, may fulfil the usual role in Pi uptake. Furthermore, it was shown that even if AtPHT1;3 contributed to the Pi uptake under Pi starvation, most of the Pi was taken up by another Pi transporter (PHT1;4), indicating that Pi uptake is not the main function of PHT1;3 or that there is, at least, an additional function. Thus, Młodzińska and Zboińska (2016) suggested an involvement of AtPHT1;3 in the regulation of the Pi translocation into the xylem. Since Pi may function as a signaling molecule in sensing Pi starvation (Chiou and Lin, 2011), the allocation of Pi may have a great impact, particularly as it is suggested that systemic Pi-sensing is caused by events downstream of PHT1 (Ayadi et al., 2015). In relation to this, overexpression of a Pi transporter in Arabidopsis (AtPHT1;5) and rice (OsPHT1;1) increased root hair number and length independently of the P supply and specifically under sufficient Pi conditions, respectively, which was thought to be a consequence of an altered Pi distribution or a disorder of signaling pathways (Młodzińska and Zboińska, 2016).

**Protein kinases** are another important group of regulators. They can regulate protein activity by transferring phosphate groups to proteins. Many protein kinases were specifically upregulated in Bale under Pi deficiency. For a selection, we used the paper of Lan et al. (2013), where the authors generated co-expression networks to identify protein kinases potentially involved in the Pi deficiency-induced root hair development. A central role in this process was predicted for the relative of *BcMRIL* in Arabidopsis, because it exhibited many connections to other protein kinases in the co-expression network (high edge count) (Lan et al., 2013). Recently, *AtMRIL* was shown to regulate the root hair elongation positively by acting downstream of the FER receptor and controlling the cell wall integrity in tip growing cells (Boisson-Dernier et al., 2015).

The **ROH GTPases** also control a variety of signaling pathways. They not only act as regulators of the actin cytoskeleton, but can, amongst others, also affect cell polarity, gene expression and vesicle transport (Etienne-Manneville and Hall, 2002; Scheffzek and Ahmadian, 2005). *BcROPGEF1* encodes for an activator of ROPs and it was shown for its relative in Arabidopsis, *AtROPGEF4*, that the activation was performed via the interaction with the FER receptor (Duan et al., 2010). Furthermore, *AtROPGEF4* seems to regulate root hair elongation negatively (Won et al., 2009; Lin et al., 2011; Huang et al., 2013). However, while Lin et al. (2011) observed longer root hairs in a knockout mutant of *AtROPGEF4* only under deficient Pi conditions, which indicates a specific role in the Pi deficiency-induced root hair elongation, Huang et al. (2013) concluded that *AtROPGEF4* is exclusively important for the developmental and not the environmental root hair growth regulation.

Two isoforms annotated to *AtLRL3* encoding a bHLH **transcription factor** were also investigated by knockout. *AtLRL3* is a functional paralog of *RSL4* and is necessary for a proper root hair elongation (Bruex et al., 2012; Salazar-Henao et al., 2016). Furthermore, *AtLRL3* was upregulated in response to Pi deficiency (Salazar-Henao and Schmidt, 2016).

References

Ayadi, A., David, P., Arrighi, J.-F., Chiarenza, S., Thibaud, M.-C., Nussaume, L., et al. (2015). Reducing the genetic redundancy of Arabidopsis PHOSPHATE TRANSPORTER1 transporters to study phosphate uptake and signaling. *Plant Physiol.* 167, 1511–1526. doi: 10.1104/pp.114.252338

Bibikova, T. N., Blancaflor, E. B., and Gilroy, S. (1999). Microtubules regulate tip growth and orientation in root hairs of *Arabidopsis thaliana*. *Plant J.* 17, 657–665. doi: 10.1046/j.1365-313X.1999.00415.x

Bibikova, T. N., Zhigilei, A., and Gilroy, S. (1997). Root hair growth in *Arabidopsis thaliana* is directed by calcium and an endogenous polarity. *Planta* 203, 495–505. doi: 10.1007/s004250050219

Boisson-Dernier, A., Franck, C. M., Lituiev, D. S., and Grossniklaus, U. (2015). Receptor-like cytoplasmic kinase MARIS functions downstream of CrRLK1L-dependent signaling during tip growth. *Proc. Natl. Acad. Sci. U S A* 112, 12211–12216. doi: 10.1073/pnas.1512375112

Bruex, A., Kainkaryam, R. M., Wieckowski, Y., Kang, Y. H., Bernhardt, C., Xia, Y., et al. (2012). A gene regulatory network for root epidermis cell differentiation in Arabidopsis. *PLoS Genet.* 8, e1002446. doi: 10.1371/journal.pgen.1002446

Campbell, L., and Turner, S. R. (2017). A comprehensive analysis of RALF proteins in green plants suggests there are two distinct functional groups. *Front. Plant Sci.* 8, 37. doi: 10.3389/fpls.2017.00037

Chiou, T.-J., and Lin, S.-I. (2011). Signaling network in sensing phosphate availability in plants. *Annu. Rev. Plant Biol.* 62, 185–206. doi: 10.1146/annurev-arplant-042110-103849

Czechowski, T., Stitt, M., Altmann, T., Udvardi, M. K., and Scheible, W.-R. (2005). Genome-wide identification and testing of superior reference genes for transcript normalization in Arabidopsis. *Plant Physiol.* 139, 5–17. doi: 10.1104/pp.105.063743

Duan, Q., Kita, D., Li, C., Cheung, A. Y., and Wu, H.-M. (2010). FERONIA receptor-like kinase regulates RHO GTPase signaling of root hair development. *Proc. Natl. Acad. Sci. U S A* 107, 17821–17826. doi: 10.1073/pnas.1005366107

Etienne-Manneville, S., and Hall, A. (2002). Rho GTPases in cell biology. *Nature* 420, 629–635. doi: 10.1038/nature01148

Haruta, M., Sabat, G., Stecker, K., Minkoff, B. B., Sussman, M. R., and (Keine Angabe) (2014). A peptide hormone and its receptor protein kinase regulate plant cell expansion. *Science* 343, 408–411. doi: 10.1126/science.1244454

Huang, G.-Q., Li, E., Ge, F.-R., Li, S., Wang, Q., Zhang, C.-Q., et al. (2013). Arabidopsis RopGEF4 and RopGEF10 are important for FERONIA-mediated developmental but not environmental regulation of root hair growth. *New Phytol.* 200, 1089–1101. doi: 10.1111/nph.12432

Johnson, K. L., Jones, B. J., Bacic, A., and Schultz, C. J. (2003). The fasciclin-like arabinogalactan proteins of Arabidopsis. A multigene family of putative cell adhesion molecules. *Plant Physiol.* 133, 1911–1925. doi: 10.1104/pp.103.031237.

Jun, L., and Xiaoming, W. (2012). Genome-wide identification, classification and expression analysis of genes encoding putative fasciclin-like arabinogalactan proteins in Chinese cabbage (*Brassica rapa* L.). *Mol. Biol. Rep.* 39, 10541–10555. doi: 10.1007/s11033-012-1940-1

Lan, P., Li, W., and Schmidt, W. (2013). Genome-wide co-expression analysis predicts protein kinases as important regulators of phosphate deficiency-induced root hair remodeling in Arabidopsis. *BMC Genomics* 14, 210. doi: 10.1186/1471-2164-14-210

Lin, W.-D., Liao, Y.-Y., Yang, T. J. W., Pan, C.-Y., Buckhout, T. J., and Schmidt, W. (2011). Coexpression-based clustering of Arabidopsis root genes predicts functional modules in early phosphate deficiency signaling. *Plant Physiol.* 155, 1383–1402. doi: 10.1104/pp.110.166520

Ma, Y., Yan, C., Li, H., Wu, W., Liu, Y., Wang, Y., et al. (2017). Bioinformatics prediction and evolution analysis of arabinogalactan proteins in the plant kingdom. *Front. Plant Sci.* 8, 66. doi: 10.3389/fpls.2017.00066

Marzec, M., Szarejko, I., and Melzer, M. (2015). Arabinogalactan proteins are involved in root hair development in barley. *J. Exp. Bot.* 66, 1245–1257. doi: 10.1093/jxb/eru475

Młodzińska, E., and Zboińska, M. (2016). Phosphate uptake and allocation - a closer look at *Arabidopsis thaliana* L. and Oryza sativa L. *Front. Plant Sci.* 7, 1198. doi: 10.3389/fpls.2016.01198

Morato do Canto, A., Ceciliato, P. H.O., Ribeiro, B., Ortiz Morea, F. A., Franco Garcia, A. A., Silva-Filho, M. C., et al. (2014). Biological activity of nine recombinant AtRALF peptides: Implications for their perception and function in Arabidopsis. *Plant Physiol. Biochem.* 75, 45–54. doi: 10.1016/j.plaphy.2013.12.005

Mudge, S. R., Rae, A. L., Diatloff, E., and Smith, F. W. (2002). Expression analysis suggests novel roles for members of the Pht1 family of phosphate transporters in Arabidopsis. *Plant J.* 31, 341–353. doi: 10.1046/j.1365-313X.2002.01356.x

Murphy, E., and De Smet, I. (2014). Understanding the RALF family: a tale of many species. *Trends Plant Sci.* 19, 664–671. doi: 10.1016/j.tplants.2014.06.005

Petersen, T. N., Brunak, S., Heijne, G. von, and Nielsen, H. (2011). SignalP 4.0: Discriminating signal peptides from transmembrane regions. *Nat. Methods* 8, 785–786. doi: 10.1038/nmeth.1701

Pierleoni, A., Martelli, P. L., and Casadio, R. (2008). PredGPI: A GPI-anchor predictor. *BMC Bioinformatics* 9, 392. doi: 10.1186/1471-2105-9-392

Salazar-Henao, J. E., and Schmidt, W. (2016). An inventory of nutrient-responsive genes in Arabidopsis root hairs. *Front. Plant Sci.* 7, 237. doi: 10.3389/fpls.2016.00237

Salazar-Henao, J. E., Velez-Bermudez, I. C., and Schmidt, W. (2016). The regulation and plasticity of root hair patterning and morphogenesis. *Development* 143, 1848–1858. doi: 10.1242/dev.132845

Scheffzek, K., and Ahmadian, M. R. (2005). GTPase activating proteins: structural and functional insights 18 years after discovery. *Cell. Mol. Life Sci.* 62, 3014–3038. doi: 10.1007/s00018-005-5136-x

Seifert, G. J., and Roberts, K. (2007). The biology of arabinogalactan proteins. *Annu. Rev. Plant Biol.* 58, 137–161. doi: 10.1146/annurev.arplant.58.032806.103801

Steibel, J. P., Poletto, R., Coussens, P. M., and Rosa, G. J.M. (2009). A powerful and flexible linear mixed model framework for the analysis of relative quantification RT-PCR data. *Genomics* 94, 146–152. doi: 10.1016/j.ygeno.2009.04.008

Won, S.-K., Lee, Y.-J., Lee, H.-Y., Heo, Y.-K., Cho, M., and Cho, H.-T. (2009). Cis-element- and transcriptome-based screening of root hair-specific genes and their functional characterization in Arabidopsis. *Plant Physiol.* 150, 1459–1473. doi: 10.1104/pp.109.140905

Wu, J., Kurten, E. L., Monshausen, G., Hummel, G. M., Gilroy, S., and Baldwin, I. T. (2007). NaRALF, a peptide signal essential for the regulation of root hair tip apoplastic pH in *Nicotiana attenuata*, is required for root hair development and plant growth in native soils. *Plant J.* 52, 877–890. doi: 10.1111/j.1365-313X.2007.03289.x

Wymer, C. L., Bibikova, T. N., and Gilroy, S. (1997). Cytoplasmic free calcium distributions during the development of root hairs of *Arabidopsis thaliana*. *Plant J.* 12, 427–439. doi: 10.1046/j.1365-313X.1997.12020427.x
